# Topology-inclusive aerodynamic shape optimisation using a cellular automata parameterisation

**DOI:** 10.1007/s00158-024-03916-6

**Published:** 2025-01-30

**Authors:** M. J. Wood, T. C. S. Rendall, C. B. Allen, L. J. Kedward, N. J. Taylor, J. Fincham, N. E. Leppard

**Affiliations:** 1 Department of Aerospace Engineering, University of Bristol, Bristol, BS8 1TR UK; 2 MBDA UK Ltd, Bristol, BS34 7QS UK; 3 BAE Systems (Operations) Ltd, Bristol, UK

**Keywords:** Optimisation, Parameterisation, Cellular automata, Aerodynamic topology optimisation

## Abstract

A novel geometry parameterisation method constructed from a volume-of-solid driven cellular automata is presented. The method is capable of describing complex geometry of arbitrary topology using a set of volume-of-solid parameters applied to a geometry control mesh. This is done by approximating the smooth geometry of minimum surface area subject to a set of localised constraints on contained volume defined by both the control mesh and volume-of-solid parameters. Localised control mesh refinement is possible through splitting of control mesh cells to provide additional degrees of freedom where necessary. The parameterisation is shown to reconstruct over 98% of a library of aerofoil geometries to within a standard wind tunnel-equivalent geometric tolerance, and to recover known analytical optima in supersonic flow. Using gradient-free optimisation methods, the parameterisation is then shown to construct aerodynamic geometries consisting of multiple objects to package a set of existing geometries. Finally, the parameterisation is used to construct an optimal supersonic multi-body geometry with less than half the drag of the equivalent volume optimal single body.

## Introduction

In the design of aircraft and machinery where aerodynamics is of significant importance, shape and topology optimisation are becoming increasingly important tools to achieve ambitious performance targets. Shape and topology optimisation of geometry for aerodynamic objectives can be a complex process, as it requires the seamless integration of methods for geometry representation or deformation, with tools to evaluate the aerodynamic performance of these geometries, all while the geometry is manipulated by an optimisation algorithm. To achieve this, some form of geometry parameterisation is required. This is a method that translates a set of values that are controlled by the optimisation algorithm, the design variables, into a numerical description of geometry, such as a mesh. For the design and optimisation of geometries driven by aerodynamic objectives for which changes in topology may be desirable, limited choices of geometry parameterisation exist. Many parameterisation methods designed for aerodynamic shape optimisation do not naturally allow for changes in the topology of the geometry they represent with only changes to their design variables, whereas many topology-inclusive parameterisation methods designed for structural optimisation have properties that limit their utility for optimisation driven by aerodynamic objectives. Consequently, the development of new topology-inclusive geometry parameterisation methods suitable for representing aerodynamic geometries is critical to facilitating topology-inclusive shape optimisation driven by aerodynamic objectives.

There however exist already many topology-inclusive parameterisation methods designed for the optimisation of structural geometries. Many of these methods are based in some way on the SIMP method, derived by Bendsøe and Kikuchi (1988). This method represents geometry as a discretised density field, with parameters $\rho \in [0,1]$ controlling an interpolation between the properties of material and empty space within discrete volumes in the design domain. This type of method performs well for structural optimisation, as the smoothness properties of surfaces are often unimportant compared to the physical placement of material, and the objective function often depends on volumetric properties such as stress, such that objective sensitivities exist everywhere within the design domain. This allows efficient gradient-based optimisation algorithms to be used to perform topology-inclusive structural optimisation, even with millions of design variables, Aage et al. (2017).

For aerodynamics-based design objectives however, there are two important differences. First, many of these objectives, such as pressure drag for example, are defined as a function of properties on surfaces, resulting in the fact that objective sensitivities exist only on these surfaces, not everywhere within the volume of the design domain as is the case for many structural design objectives. Consequently, there likely exist many spurious local minima within topology-inclusive aerodynamic design spaces. This is supported by the fact that even some far simpler aerodynamic shape optimisation cases with fixed topology exhibit multi-modal behaviour, Poole et al. (2018), likely for similar reasons. Consequently, to reliably overcome these spurious local minima and allow exploration of multiple topologies, gradient-free optimisation algorithms must be utilised. However as aerodynamic objectives are often computationally expensive to evaluate, until recently this approach has been impractical. And second, the smoothness properties of surfaces are often of at least equivalent importance for the aerodynamic properties of a geometry as the positioning of the surfaces themselves.

Consequently, many previous attempts at topology-inclusive optimisation for aerodynamic objectives have utilised finite-element fluid models and porosity boundary conditions to implement gradient-based optimisation methods similar to the SIMP method used for structural optimisation. Examples include work by Borrvall and Petersson (2002), Gersborg-Hansen et al. (2005) and Pingen et al. (2009). However, due to the reliance of these methods on a finite-element fluid model and porosity boundary conditions, they are only applicable to low speed incompressible flows, significantly limiting their utility. In addition, parameterisations similar to the SIMP method suffer from two further issues that impact their utility for optimisation driven by aerodynamic objectives. First, due to their density-based formulation they do not define a smooth solid surface, making them difficult to integrate with conventional finite-volume fluid models. Second, due to the often large number of design variables, these parameterisation methods require, their integration with gradient-free optimisation algorithms is often computationally impractical. Thus, to enable topology-inclusive aerodynamic shape optimisation across a wider range of flow conditions, new geometry parameterisations of lower dimensionality that are more suitable for representing aerodynamic geometry must be defined.

There do however exist many geometry parameterisation methods designed specifically for aerodynamic shape optimisation. These can broadly be sorted into one of two classes, constructive or deformative. Constructive methods define geometry completely from a set of parameters, whereas deformative methods seek only to define deformations to some existing geometry.

Constructive methods such as B-splines, Sobieczky (1997), class shape transforms, Kulfan (2008) or PARSEC, Sobieczky (1999) define fully the geometry from a set of variables alone. Many other methods of this type, including methods that define geometry through combinations of mode shapes defined by singular value decomposition of a geometry library: Poole et al. (2015a), Poole et al. (2015b), Allen et al. (2018), or methods that define geometry as a subdivision surface: Masters et al. (2016), Wood et al. (2022b), have been shown to perform well across many different aerodynamic shape optimisation cases. However, most constructive methods are restricted such that the topology of the geometry they represent is fixed, and cannot be modified by changing the design variables controlling the geometry alone. This restricts their ability to explore different topologies during an optimisation. Recent work by Payot et al. (2019a) and Payot et al. (2019b) has resulted in the development of a constructive method where the topology of the parameterised geometry can vary with only changes to the design variables. However, this method has complexities in its implementation, especially in three dimensions, making it difficult to utilise.

Deformative methods define only perturbations to some initial geometry. As a result, they are often capable of handling the optimisation of complex geometries of arbitrary topology in both two and three dimensions, which constructive methods may struggle to represent. One type of deformative method involves applying local deformations to surfaces directly. This can take the form of a method such as that of Hicks and Henne (1977), where bump functions are applied to an initial surface to locally deform it. More recently, work by Kedward et al. (2018) presented a method of a similar type, where local deformations to geometry can be applied by manipulating the position of each vertex defining the surface of the geometry individually. Constraints are then applied to ensure the surface remains smooth. Another widely used type of deformative method is that of free form deformation Andreoli et al. (2003), Morris et al. (2008). This type of method interpolates the deformations of points placed in a volume onto the surface of some arbitrary geometry. This method has seen widespread adoption and use across many different aerodynamic shape optimisation cases due to its simplicity and ease of implementation. However, all deformative methods again come with the same restriction as most constructive methods; they lack the ability to change the topology of the geometry they deform.

Level set methods can be viewed as either constructive or deformative methods depending on the implementation. Following the implementation as described by Sethian (2009), using gradients to drive the evolution of the contour forms a deformative method. This in theory provides the capability to explore topology changes. However, as stated previously, gradient-free optimisation algorithms are likely required to explore topology changes in most aerodynamic shape optimisation cases due to the non-existence of objective sensitivities away from geometry surfaces leading to the existence of spurious locally optimal solutions. However, integrating a gradient-free optimisation algorithm, such as differential evolution, Storn and Price (1997) or particle swarm, Kennedy and Eberhart (1995), with a level set method can be difficult. This is because the design variables output by these optimisation algorithms describe a position in the design space directly, not a change in position that improves the objective, as is output by gradient-based optimisation algorithms. Consequently, to integrate these types of optimisation algorithm with level set parameterisations, it is often necessary to use them to explicitly define a function directly with the design variables, that a level set can then be taken from to define the geometry. An example of such a scheme can be found in the method derived by Pingen et al. (2009).

Overall, it appears that constructive methods with very general design variables, such as the volume-of-solid type method presented by Payot et al. (2019a) appear to be a strong class of geometry parameterisation for topology inclusive aerodynamic shape optimisation. This is due to the smooth nature of the surfaces they define, the simplicity of their integration with gradient-free optimisation algorithms, and their efficient parameterisation of changes in topology. Thus, this work builds on previous efforts by the authors Wood et al. (2022a) to create a constructive volume-of-solid parameterisation method. Similar to the work of Payot et al. (2019a), this method builds surfaces of approximately minimum area, subject to a set of spatially distributed contained volume constraints defined on control regions within the design domain. To achieve this, this new method defines geometry in two stages. The first stage uses a cellular automata scheme (Wolfram 1983), Gardner (1970), to classify volumetric sub-regions within each control region in the design domain as inside or outside of geometry. Subsequently in the second stage, using a contouring method, an explicit surface is constructed as a level set on the solution of the cellular automata, and smoothing is applied to the surface. This results in a solid smooth surface suitable for the parameterisation of aerodynamic geometries. The method is designed such that it is simple to implement, efficient, and reliable in both two and three dimensions. This work presents the formulation and development of this method, along with its application to a set of test cases involving both gradient-based and gradient-free aerodynamic shape optimisation.

## Cellular automata volume-of-solid parameterisation

In this section, the formulation of the novel ‘cellular automata volume-of-solid’ (CAVoS) geometry parameterisation method is presented. This method defines geometry using a cellular automata scheme controlled by a set of volume-of-solid parameters $v_t \in [0,1]$, each associated with one cell $C_i$ in a ‘control mesh’ that tiles the parameterisation domain. This control mesh is used to define where geometry exists across a set of separate domains $\Omega _i$. Each volume-of-solid parameter $v_{ti}$ specifies the fraction ∈[0,1] of the domain $\Omega _i$ defined by its corresponding control mesh cell $C_i$ that is contained within the geometry.

**Fig. 1:**
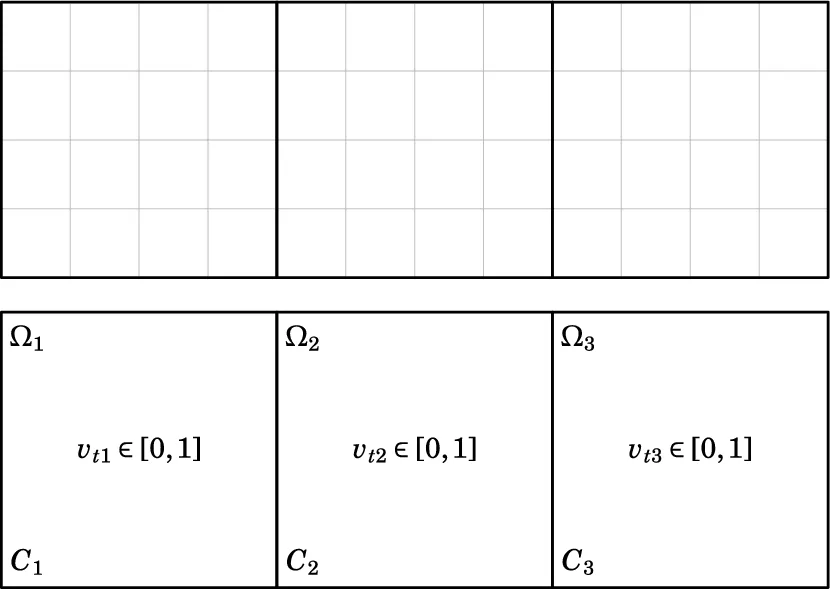
A control mesh consisting of three control cells, each containing a 4×4 cell sub-grid

Within each cell of the control mesh exists a sub-grid, upon which the cellular automata operates. The sub-grid within each control cell is an independent entity which can be represented as a matrix ${\textbf{C}}_i$. An example control mesh and its corresponding sub-grids is shown in Fig. 1.

Geometry is constructed by running a cellular automata scheme on the sub-grids within all control cells simultaneously. This sets the state ρ of each sub-grid cell to specify if each is empty space ρ=0, or contained within geometry ρ=1. An explicit geometry surface is then defined as a mesh of a level set of ρ at $0< \rho _s < 1$, and is smoothed using a volume constrained surface area minimising smoothing algorithm.

1 $$\begin{aligned} \begin{aligned} \text {min }&: A_s \\ \text {subject to }&: \mathbf {v_c} - \mathbf {v_t} = 0 \\ \end{aligned} \end{aligned}$$

The cellular automata is derived such that the distribution of ρ defined by its steady-state solution describes a geometry of approximately minimum surface area $A_s$, subject to equality constraints on the geometry contained volume fractions $\mathbf {v_c}$ of each control cell, defined by each cell’s corresponding volume-of-solid parameter $\mathbf {v_t}$, Eq. 1.

**Fig. 2:**
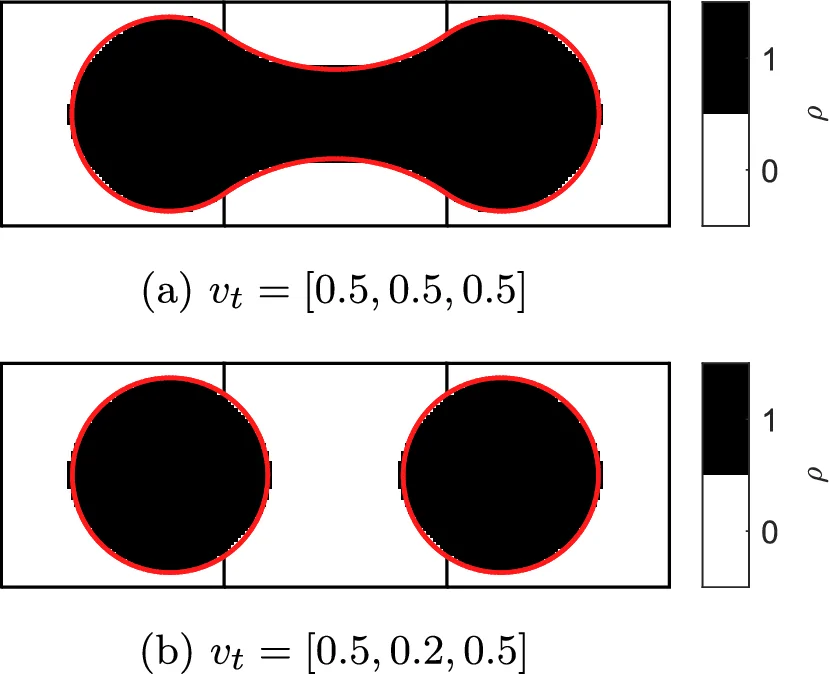
Example geometries constructed with the CAVoS parameterisation on three control cells with two sets of different $v_t$, each resulting in a different topology. The red line shows the smooth parameterised surface and the black and white shading represents the state ρ of each sub-grid cell

Due to this definition of geometry as the result of a cellular automata operating on volumetric units, no assumptions are made about, or restrictions placed upon, the topology of this geometry. Thus, parameterisation of arbitrary topologies and changes in topology of the parameterised geometry can be achieved simply through changes in each volume-of-solid parameter $v_t$, Fig. 2.

The remainder of this section describes each component of this parameterisation. First, the structure of the control mesh and each sub-grid is defined. The methods by which each sub-grid interacts to allow definition of geometry spanning multiple control cells are also discussed. Subsequently, the cellular automata scheme is derived, and some of its properties investigated. Finally, methods to construct and smooth an explicit surface mesh from the result of the cellular automata are outlined.

### Structure of the control mesh and sub-grids

The control mesh defines each spatial region that corresponds to each volume-of-solid parameter $v_t$. This mesh covers the entire domain in which the scheme can construct geometry and consists of $N_{ctr}$ control cells, each with one corresponding volume-of-solid parameter $v_{ti}$. The control mesh is structured in a quadtree/octree format such that some control cells may be refined to represent smaller regions of physical space.

**Fig. 3:**
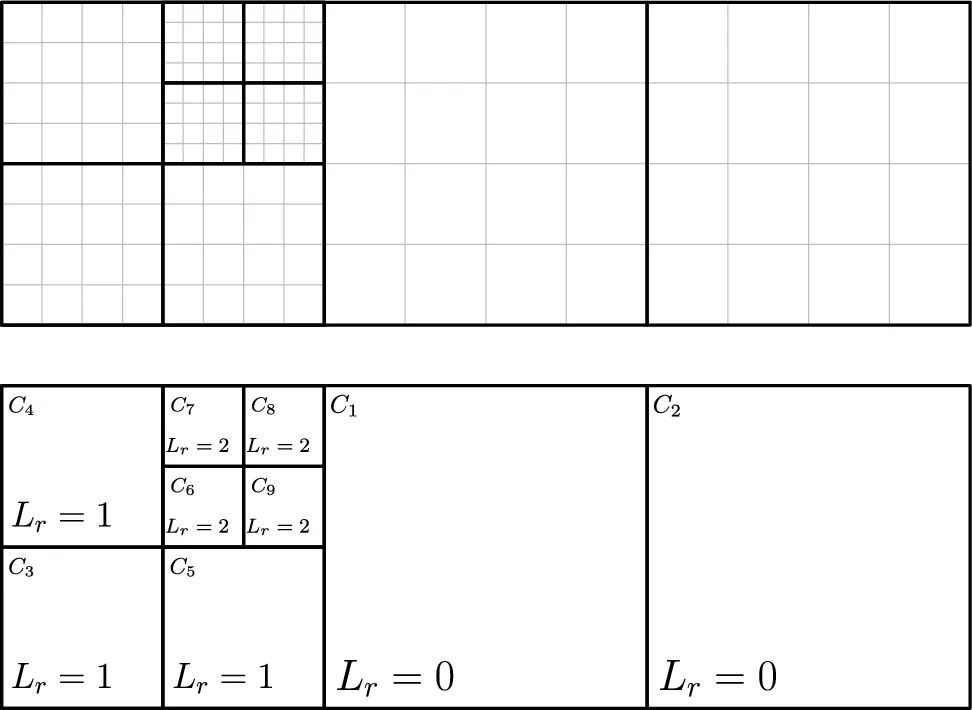
A control mesh of $N_{ctr} = 9$ control cells with refinement levels $L_r$ of zero to two, each containing a sub-grid of $R_c = 4$

Each control cell has an associated refinement level $L_r$, starting from zero and increasing by one upon each quadtree/octree refinement. An example control mesh consisting of 9 control cells of refinement levels $L_r = 0 \rightarrow L_r = 2$ is shown in Fig. 3.

**Fig. 4:**
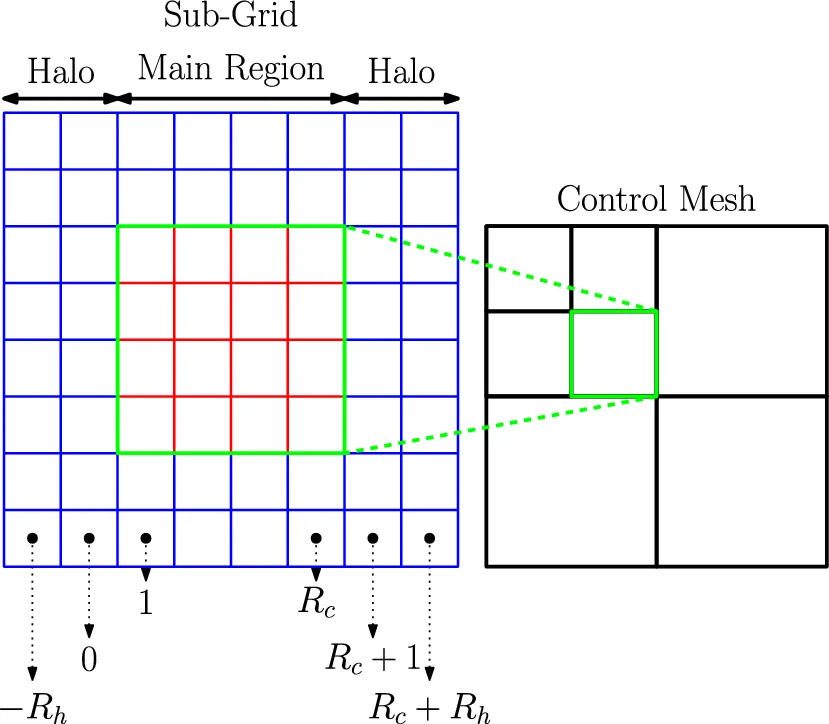
A control mesh with an expanded view of the sub-grid within one control cell with $R_c = 4$ and $R_h = 2$. Within the sub-grid, red indicates the main region and blue the halo region. The sub-grid ${\textbf{C}}$ is defined by the entire region encompassing both blue and red regions

Within each control cell exists an independent sub-grid. Each sub-grid is represented by a matrix ${\textbf{C}}_i$. This matrix contains two zones, a main region and a halo (boundary) region. The main region represents the sub-grid cells within the control cell *i*, whereas the sub-grid cells in the halo region act as boundary conditions for the main region. The main region is of size $R_c \in 2^n$ cells, whereas the halo region is of size $R_h$ cells, as in Fig. 4.

The state ρ of each sub-grid cell in the halo region of control cell *i* is set by interpolating the states of sub-grid cells from the main regions of adjacent control cells *j*. This interpolation permits the cellular automatas within the sub-grids of separate adjacent control cells *i* and *j* to influence each other though changing each others boundary conditions. This allows multiple cellular automatas to act together across the entire control mesh to construct geometry spanning multiple control cells.

**Fig. 5:**
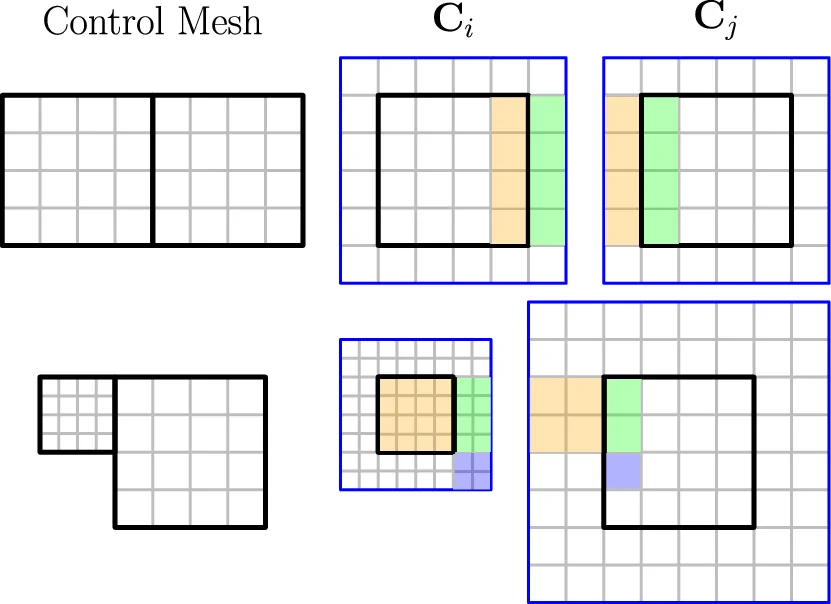
Linked sections of the halo and internal regions for two configurations of two control cells with $R_c = 4$. Each configuration of varying $L_r$ has the minimum possible $R_h$. The pairs of sections for populating the halo region in ${\textbf{C}}_i$ are shaded green or blue, whereas the pairs of sections for populating the halo region in ${\textbf{C}}_j$ are shaded orange

To interpolate the cell states within the halo region of each control cell *i*, the structure of the control mesh can be used to link sections of the halo region of control cell *i* to sections of the main region of adjacent control cells *j*. This mapping links sections of the halo region of control cell *i* to the main region sections in control cell *j* that they overlap, Fig. 5. If there is a difference in refinement level between control cells *i* and *j*, there will be a one to many or many to one mapping from each cell in the linked main region section to the cells in the halo region. This necessitates either an expansion or averaging operation, respectively, to interpolate or approximate the cell states within the halo region.

To ensure the influence of each control cell is only transferred to its direct neighbours through their halo regions, limits are imposed on the permissible difference in refinement levels between adjacent control cells. The bound in the case $L_{ri} < L_{rj}$ arises from ensuring the halo region of control cell *i* is no larger than the main region of control cell *j*. The bound in the case $L_{ri}> L_{rj}$ arises from ensuring the halo region of control cell *i* is no smaller than one sub-grid cell in main region of control cell *j*. Taking both into account, the maximum permissible change in refinement levels between two adjacent control cells is given by Eq. 2.

2 $$\begin{aligned} \max \left( \Delta L_r \right) = \min {\left\{ \begin{array}{ll} \left| \log _2\left( \frac{R_c}{R_h}\right) \right| \\ \\ \left| \log _2\left( \frac{1}{R_h}\right) \right| \\ \end{array}\right. } \end{aligned}$$

### Structure of the cellular automata

A cellular automata is an iterative scheme where at each iteration the state of every cell in a grid is updated as a function of the states of other nearby cells. Each cell can have two states, zero and one. Using a small set of simple rules, these schemes can give rise to complex emergent behaviour, Wolfram (1983), Gardner (1970). For this application, a cellular automata scheme is used to define geometry by setting the state of each cell to specify if it is inside or outside of the geometry. To this end, cells of state zero (ρ=0) are used to describe regions of empty space, and cells of state one (ρ=1) are used to describe regions within the geometry. Thus, the boundary between all cells of ρ=1 and ρ=0 defines the surface of the geometry.

To construct shapes that are ‘useful’ for the parameterisation of aerodynamic geometries, a similar formulation to the method of Payot et al. (2019a) is followed, where the parameterisation defines geometry of approximately minimum surface area, subject to a set of localised volume constraints defined within each control cell. Thus, to achieve this in the context of this work, a cellular automata must be derived that minimises the area of the boundary between ρ=1 and ρ=0 cells. This is done by exploiting an equivalence between the average number of ρ=1 neighbours of ρ=1 cells, and the area of the boundary between the ρ=1 and ρ=0 cells, equivalent to the surface area of the geometry described by the set of all ρ=1 cells.

**Fig. 6:**
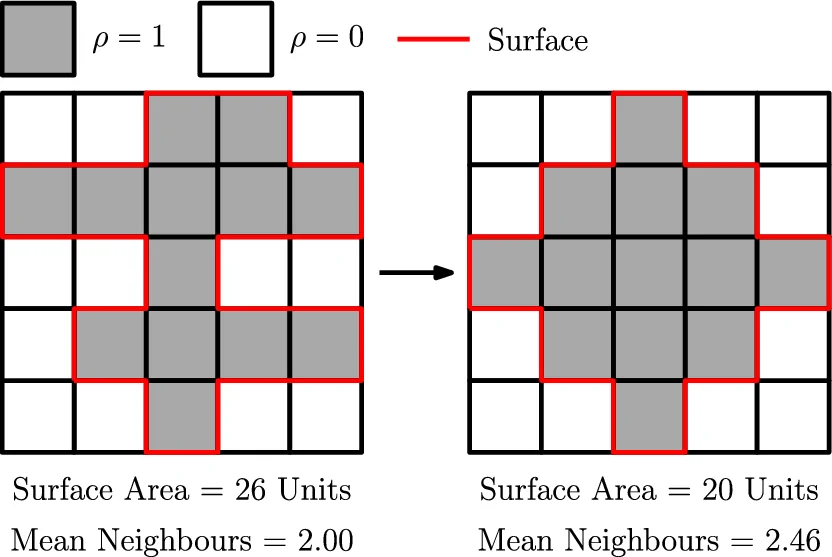
Two arrangements of the same number of ρ=1 cells showing that increasing the average number of ρ=1 neighbours of ρ=1 cells reduces the area of the boundary between ρ=1 and ρ=0 cells

To demonstrate this, Fig.6 shows two distributions of the same number of ρ=1 cells, where it is evident that when arranged such that the average number of ρ=1 neighbours of ρ=1 cells is larger, the area of the boundary between ρ=1 and ρ=0 cells is reduced. Additionally, it can be noted that the volume of a geometry described in this way, by some arrangement of ρ=1 cells, is given simply by the total number of ρ=1 cells. Thus, to design a cellular automata to minimise the surface area of geometry described by ρ=1 cells subject to some form of constraint on its volume, the cellular automata must work to maximise the average number of ρ=1 neighbours of all ρ=1 cells, while also controlling the total number of ρ=1 cells.

The remainder of this section thus presents the derivation of such a cellular automata scheme. This is done using a ‘habitability function’ *h*. This controls where ρ=1 cells can exist, such as to minimise the surface area of the geometry they describe, and is a function of the state of all cells within a local neighbourhood. Using this function, a set of rules are defined that govern how to iteratively change the state of the sub-grid cells within every control cell, as a function of only *h* and the volume constraint within each control cell, to minimise the surface area of the geometry described by the ρ=1 cells, while adhering to the volume constraints within each control cell.

### The habitability function

The habitability function *h* has a value at each cell within the main region of the sub-grid of each control cell. As with the cell states within each control cell ${\textbf{C}}$, this can be represented as a matrix ${\textbf{H}}$ of the same size as the main region of the sub-grid in each control cell.

To define *h*, the example of Fig. 6 can be considered. From this it is evident that a cellular automata scheme that eliminates ρ=1 cells without many ρ=1 neighbours, and replaces them with an equal number of ρ=1 cells that have a larger number of ρ=1 neighbours will reduce the surface area of the geometry they describe, without changing its volume. Thus, to derive such a scheme, *h* was defined such as to be large where there are already many ρ=1 cells, and small where there are many ρ=0 cells.

Using such an *h*, a cellular automata can be defined where cells are set to ρ=1 where *h* is ‘large’, and to ρ=0 where *h* is ‘small’. As *h* is larger in regions with more ρ=1 cells, this creates a feedback loop forcing all ρ=1 cells to cluster together to maximise their number of ρ=1 neighbours, minimising the surface area of the geometry they describe. Consequently, a natural choice to define such an *h* is that its value at each sub-grid cell, $h_i$, be defined by the normalised sum of the states of all cells within the Moore neighbourhood of size $N_r$ surrounding each sub-grid cell. In two dimensions, this is given by Eq. 3. This results in $h_i = 1$ if all cells within this region are ρ=1, $h_i = 0$ if all cells are ρ=0, and values $h_i \in [0,1]$ for any configuration in between.

3 $$\begin{aligned} {\textbf{H}}(i_x,i_y) = h_i = \sum _{i_y-N_r}^{i_y+N_r} \sum _{i_x-N_r}^{i_x+N_r} \frac{{\textbf{C}} (i_x,i_y)}{(2N_r + 1)^2} \end{aligned}$$

To efficiently evaluate Eq. 3, it can be rewritten as a dimensionally separable convolution. First, Eq. 3 can be rewritten as the equivalent Eq. 4, with $\mathbf {K_1}$ a square kernel matrix of size $2N_r + 1$, populated with all ones. As $\mathbf {K_1}$ is square and symmetric, this can be evaluated as a set of *n* recursive one-dimensional convolutions in *n* dimensions.

4 $$\begin{aligned} {\textbf{H}}= & \frac{{\textbf{C}}*\mathbf {K_1}}{(2N_r + 1)^n} \end{aligned}$$

5 $$\begin{aligned} h^k_{i}= & {\left\{ \begin{array}{ll} \sum _{j=-N_r}^{N_r} h^{k-1}_j \text {, if } i = 1\\ \\ h^{k}_{i-1} + h^{k-1}_{i+N_r} - h^{k-1}_{i-N_r-1} \text {, else} \\ \end{array}\right. } \end{aligned}$$

**Fig. 7:**
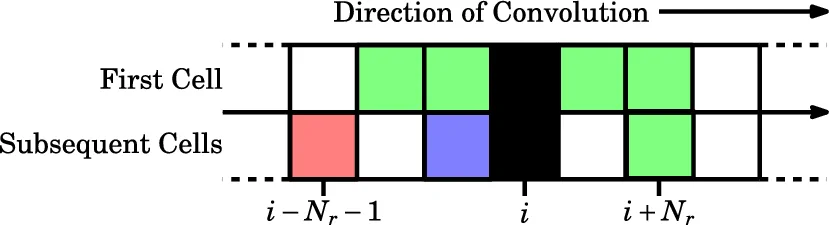
The convolution process for populating $h^k_i$ for cell *i* along each dimension *k* for $N_r = 2$. Green corresponds to addition from $h^{k-1}$, blue to addition from $h^k$ and red to subtraction from $h^{k-1}$

This process is described by Eq. 5 (Fig. 7), which is applied recursively *n* times in *n* dimensions. For the first dimension k=1, $h_i^0$ are the sub-grid cell states $c_i$ within the control cell matrix ${\textbf{C}}$, and at the final dimension, k=n, $h_i^n$ are the habitability values at each sub-grid cell within the control cell, the entries in the matrix ${\textbf{H}}$. The dimension Eq. 5 is applied to is changed at each recursion.

For example, in two dimensions, first Eq. 5 is applied in the $i_x$ direction, then is applied to the result of this in the $i_y$ direction. In three dimensions, Eq. 5 would subsequently then be applied to the result of the $i_y$ direction convolution in the $i_z$ direction. In *n* dimensions, this method constructs the convolution in 3*n* integer additions for each control cell internal sub-grid cell, with an additional $2N_r + 1$ integer additions for the first cell along each dimension. This results in a scheme more efficient in terms of operations than directly evaluating Eq. 3, which would result in $(2N_r + 1)^n R_c^n$ integer additions in *n* dimensions, and avoids the slow complex-complex multiplications of a fast Fourier transform convolution.

**Fig. 8:**
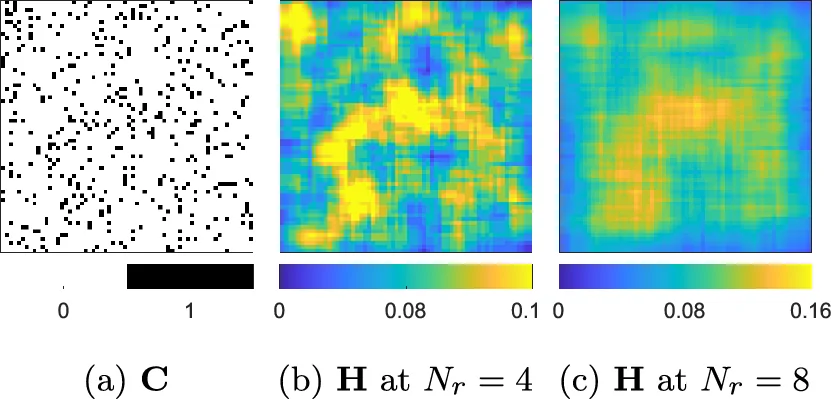
The habitability function evaluated on an $R_c = 64$ sub-grid of randomly distributed ρ=1 and ρ=0 cells at different values of $N_r$. All halo region sub-grid cell states were set to ρ=0

Fig. 8 shows an example of ${\textbf{H}}$ evaluated using this method on a set of randomly distributed ρ=1 sub-grid cells at two values of $N_r$. It can be noted that as $N_r$ is increased, ${\textbf{H}}$ appears more diffuse with smaller regions taking larger values.

The case of the first cell $i_{x/y/z} = 1$ in Eq. 5 is where the halo regions of each control cell are utilised. Looking at this case, it can be seen that as the index *j* becomes negative, the states of the cells in the halo region of the control cell contribute to the value of $h_i$ in this first sub-grid cell along each dimension within the main region of the control cell. Thus, the cell states in the halo region of a control cell effectively act as a boundary condition on the habitability values within the control cell.

### Rules to set the sub-grid cell states

The iterative process by which the state ρ of each cell is updated is based on a set of rules that define the new state as a function of the current states of the surrounding cells. These rules are constructed on the premise that the habitability function *h*, a function only of current cell states, is assumed to drive ρ=1 cells to arrange into a shape that minimises their surface area over time. From first principles, to define a geometry as a function of only *h*, one can take a level set of this function $h_b$. This contour of the function *h* can be used to define an approximate surface position.

To translate this to a volumetric representation, the inside/outside of the surface state of each cell on the sub-grid can be sampled by comparing the value of habitability in each sub-grid cell $h_i$ to the level set value $h_b$. The state of each cell can then be set as a function of this inside/outside determination. In addition, if the value of $h_b$ is selected carefully, a desired net change in the quantity of cells of ρ=1 can be achieved. This idea thus defines the premise of the iterative scheme presented in this section; select a value of $h_b$ at each iteration such as to ensure the total quantity of cells of ρ=1 tends to a target value, while these cells arrange themselves to minimise their total surface area, driven by the local values of the habitability function.

6 $$\begin{aligned} c_i^{k+1} = {\left\{ \begin{array}{ll} 1 \text {, if: } h_i^k \ge h_b \\ 0 \text {, if: } h_i^k < h_b \\ \end{array}\right. } \end{aligned}$$

With this premise in mind, the explicit method by which the state of each cell is set at iteration *k* can be defined though Eq. 6. For each cell *i* in control cell ${\textbf{C}}$ it is set to ρ=1 at iteration k+1 if its habitability value at iteration *k* is greater than or equal to $h_b$, else it is set to ρ=0 for iteration k+1.

The selection of $h_b$ is thus critical to the performance and behaviour of the cellular automata scheme. The value chosen for $h_b$ must ensure that over time the fraction of the main region of the control cell matrix ${\textbf{C}}$ containing cells of ρ=1 tends towards the required geometry contained volume fraction specified by the relevant volume-of-solid parameter $v_{t}$. To aid this, a desirable property of $h_b$ would be that small perturbations in its value cause a net increase or decrease in the quantity of ρ=1 cells in the control cell. Thus, to select such an $h_b$, a two stage process is used. First, a value $h_0$ for $h_b$ with this property is chosen, with $h_0$ then perturbed as a function of the error in the contained volume fraction within the control cell to select $h_b$.

7 $$\begin{aligned} h_0 = {\left\{ \begin{array}{ll} \min ({\textbf{H}} \in {\textbf{C}} = 1) \text { if: } v_c> 0 \text { and } d_v \ge 0 \\ \max ({\textbf{H}} \in {\textbf{C}} = 0) \text { if: } v_c> 0 \text { and } d_v < 0 \\ \max ({\textbf{H}} \in {\textbf{C}} = 0) \text { if: } v_c = 0 \text { and } v_t> v_{min} \\ (2N_r + 1)^n + 1 \text { if: } v_{c} = 0 \text { and } v_{t} \le v_{min}\\ \end{array}\right. } \end{aligned}$$

To select $h_0$ the rules defined in Eq. 7 are proposed. The decision criteria for these rules contain only parameters relating to the contained volume fraction within a control cell; $v_c$, the current contained volume fraction, $v_t$, the target contained volume fraction (the volume-of-solid parameter for the control cell), $d_v$, the error in the geometry contained volume fraction, and $v_{min}$, the minimum sustainable volume fraction (addressed subsequently in this section). The current contained volume fraction $v_c$ in *n* dimensions can be evaluated though Eq. 8.

8 $$\begin{aligned} v_c = \frac{1}{R_c^n}\sum _{i} {\textbf{C}}(i) \end{aligned}$$

The error in volume fractions can then subsequently be defined through Eq. 9.

9 $$\begin{aligned} d_v = v_c - v_t \end{aligned}$$

Following selection of $h_0$, the value of $h_b$ is found though a perturbation of $h_0$ by a function of the volume fraction error $d_v$, Eq. 10, where $k_v$ is a constant that satisfies $k_v> 0$.

10 $$\begin{aligned} h_b = {\left\{ \begin{array}{ll} h_0 \text { if: } v_c = 0 \text { and } v_t \le v_{min}\\ h_0(1 + k_v d_v) \text { else }\\ \end{array}\right. } \end{aligned}$$

The first two rules, where $v_c> 0$, drive the main behaviour of the scheme. These select an $h_0$ with the property that a small perturbation will cause a net change in the number of ρ=1 cells. For example, if $d_v \ge 0$, the current volume fraction is too large and the first rule is selected, so $h_0$ is selected as the minimum value of any cell of ρ=1. Thus, when Eq. 10 is applied to set $h_b$, $h_b$ will be greater than $h_0$ by a factor of $1 + k_v d_v$, thus on the next iteration all cells with habitability $h_0$ and those with habitability less than or equal to $h_b$ will become ρ=0, and there will be a net decrease in the total number of ρ=1 cells, reducing the magnitude of the volume fraction error $d_v$.

Conversely if $d_v < 0$, the current volume fraction is too small and the second rule is selected. With $h_0$ then the maximum value in any ρ=0 cell, and $h_b$ less than $h_0$ by a factor of $1 + k_v d_v$, there will be a net increase in the number of ρ=1 cells, again reducing the magnitude of the volume fraction error $d_v$.

The case where $d_v = 0$ requires special attention. If as one may initially expect is ideal, no change in the number of ρ=1 cells is forced, then the scheme stalls and the geometry is not permitted to evolve to approximate the shape of minimum surface area. The scheme will just stop with whatever geometry is described by the current cell states as soon as it happens to converge on the correct contained volume fraction. A better solution is thus to activate the first rule, leading to $h_b = h_0$, then when updating the cell states all cells with $h < h_b = h_0$ become ρ=0. This forces the iterative cell state updating procedure to jump either side of the true desired geometry contained volume fraction, but still allows the geometry to evolve to minimise its surface area while approximately maintaining the target contained volume fraction constraint. This case will only lead to termination of the scheme if $d_v$ and the current cell states both best approximate the minimum surface area for the requested configuration, such that no cells change state when this first rule is activated.

The third and fourth rules perform a different task than the first two. To describe their purpose, first $v_{min}$ must be defined. The value $v_{min}$ defines what can be thought of as the minimum ‘sustainable’ geometry contained volume fraction in a control cell. To quantify this, one can consider a control cell where the only ρ=1 cells within it form an isolated group that fits completely within a region having a diameter less than or equal to $N_r + 1$ with no other ρ=1 cells within a distance of $N_r + 1$ from this objects edges. In this configuration, the habitability value $h_i$ for all ρ=1 cells within the object is the same. If the target geometry contained volume fraction described by $v_t$ is less than the geometry contained volume fraction described by this object, a perturbation will be applied such that $h_b < h_0$, where in this case $h_0$ is the value of *h* in all ρ=1 cells. Hence, within one iteration, all these cells would become ρ=0 and the geometry contained volume fraction of the control cell would drop to zero.

Hence, $v_{min}$ is effectively the smallest possible non-zero volume-of-solid parameter that the cellular automata scheme can recover, as any volume-of-solid parameter value $v_t < v_{min}$, without any influence from adjacent control cells, will result in an control cell with $v_c = 0$ despite a non-zero volume-of-solid parameter $v_t$. Consequently, $v_{min}$ can be defined in *n* dimensions as the geometry contained volume fraction described by such an object, given by Eq. 11.

11 $$\begin{aligned} v_{min} = \frac{(N_r + 1)^n}{R_c^n} \end{aligned}$$

Returning to the third rule in Eq. 7, this allows ρ=1 cells to form within a control cell containing only ρ=0 cells if the requested contained volume fraction $v_t$ is greater than $v_{min}$. The fourth rule acts to eliminate an ‘unstable’ case by forcing $v_c \rightarrow 0$ where it occurs. This unstable case arises where $v_t \le v_{min}$ and can sometimes cause an oscillation of the geometry contained volume fraction $v_c$ between zero and one. To catch and prevent this, if in a control cell $v_t \le v_{min}$ and at some point during the iterative procedure $v_c = 0$, the fourth case of Eq. 7 and thus the first case of Eq. 10 are active, thus forcing all the cells within the control cell to remain ρ=0 with $v_c = 0$.

### Cellular automata initialisation and iterations

With both the habitability function and a set of rules to set the sub-grid cell states defined, the complete cellular automata scheme can now be outlined. To initialise the sub-grid cell states on a complete control mesh, each $v_t$ can be used to construct a crude approximation to the geometry the result of the cellular automata will describe. This is most easily achieved on a control cell by control cell basis. Sub-grid cell states can be set following Eq. 12, where in control cell *i*, if $v_{ti}> 0$ all cells in the sub-grid are set to ρ=1, and conversely if $v_{ti} = 0$ then all cells are set to ρ=0.

12 $$\begin{aligned} \mathbf {C_i} = {\left\{ \begin{array}{ll} 1 \text { if: } v_{ti}> 0 \\ 0 \text { if: } v_{ti} = 0 \\ \end{array}\right. } \end{aligned}$$

This constructs a crude approximation to the geometry to the resolution of the control mesh. Following this, the iterative cellular automata scheme can be run to construct the actual geometry described by the combination of the control mesh and all $v_t$. Each iteration of this scheme consists of five stages:

1. Set the states of all sub-grid cells in the halo region of each control cell to correctly represent the influence of adjacent control cells
2. Evaluate the habitability function ${\textbf{H}}$ for each control cell from current cell states, Eq. 4
3. Evaluate $v_c$ in each control cell from the current cell states, Eq. 8
4. Select an appropriate habitability bound value $h_b$ in each control cell using Eqs. 7 & 10
5. Update the state of each sub-grid cell in every control cell using $h_b$ and Eq. 6

To ensure consistency of the scheme, it is important that stage one is completed across all control cells before the state of any sub-grid cells is updated. This ensures the influence of each control cell on each other is derived from the system at the same point in time for all control cells. In terms of implementation, one useful characteristic of this method is that each stage of this iteration process past stage one is completely independent within each control cell. This permits efficient use of parallel computing to make the process wall-time efficient for large control meshes.

### Surface construction and smoothing

To construct a smooth surface from the converged cellular automata sub-grid cell states, a surface mesh is described on a level set of the cell states. Interpreting the cell states as density values $\rho \in [0,1]$, the surface is defined as the level set of $\rho _s = 0.5$. Construction of a mesh on this level set is performed with a modified version of the dual contouring method of Ju et al. (2002). This method constructs contours by placing vertices within the cells of a mesh, and forms mesh faces by connecting vertices in adjacent cells. In this work, for efficiency and improvements in reliability, the method has been simplified. This has been done by taking the position of each cell vertex as the average of edge vertex positions instead of the solution of a quadratic optimisation process described in the original method. However, instead of linearly interpolating the contour intersection positions along sub-grid edges, these edges are instead assumed to be Bezier curves in ρ, defined by values of ρ at each edge end and the edge midpoint.

To construct the surface mesh, the sub-grid cell states ρ are first projected to the sub-grid vertices. The interpolated density value at each vertex $\rho _v$ is found as the average of the extrapolated value from all *n* surrounding sub-grid cells using the density value and gradient in each sub-grid cell, Eq. 13.

13 $$\begin{aligned} \rho _v = \frac{1}{n} \sum _{i=1}^n \rho _i + \mathbf {\nabla \rho _i} \cdot \left( \mathbf {v_v} - \mathbf {v_{ci}}\right) \end{aligned}$$

$\mathbf {v_v}$ describes the position of a vertex *v*, and $\mathbf {v_{ci}}$ the position of the midpoint of each sub-grid cell *i*. The sub-grid density gradients are also approximated at each vertex as the average of the gradient in surrounding sub-grid cells, Eq. 14. The density at edge midpoints $\rho _{em}$ is found as the average of the linear extrapolations from each vertex at the ends of the edge, and from each sub-grid cell adjacent to the edge, using the density and density gradients at each location.

14 $$\begin{aligned} \nabla \rho _v = \frac{1}{n} \sum _{i=1}^n \mathbf {\nabla \rho _i} \end{aligned}$$

To construct vertices on this contour, a three point Bezier curve in ρ is defined along each sub-grid edge within the mesh using $\rho _{v1}$, $\rho _{em}$ , and $\rho _{v2}$. For edges where the vertex densities $\rho _{v1}$ and $\rho _{v2}$ bound the surface contour $\rho _s$, the fraction *f* along the edge at which this Bezier curve intersects the surface contour $\rho _s$ can be found by solving Eq. 15 for *f*.

15 $$\begin{aligned} \rho _s = (1 - f)^2 \rho _{v1} + 2f(1 - f) \rho _{em} + f^2 \rho _{v2} \end{aligned}$$

**Fig. 9:**
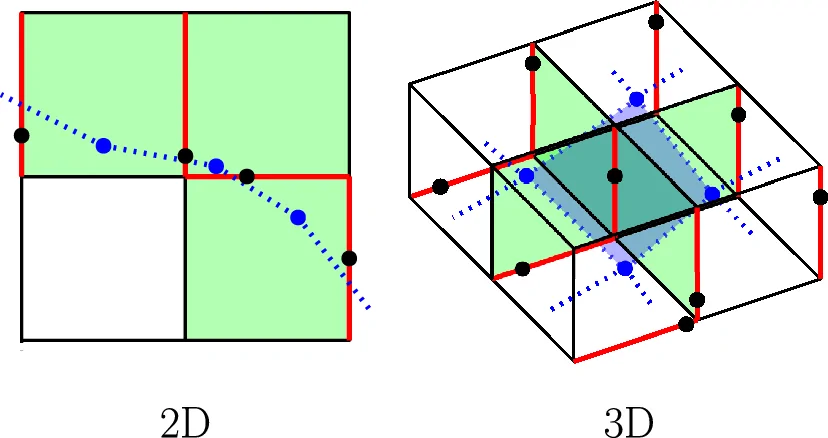
Example two- and three-dimensional sections of surface as constructed by the method described in this section. Contour intersecting sub-grid edges are shown in red, contour intersection vertices on sub-grid edges are shown in black, sub-grid cell vertices forming the surface mesh are shown in blue, and edges forming the surface mesh are shown as blue dashed lines. In 3D, the surface mesh face surrounding the central sub-grid edge is highlighted in blue

The physical solution is whichever of the two values of *f* where f∈[0,1]. Subsequently, each sub-grid cell that contains edges that intersect the surface contour has a vertex placed within the cell at the average position of all edge-contour intersections within the cell. To construct the surface mesh faces, these vertices placed in sub-grid cells are connected across each contour intersecting edge. In two dimensions, this forms edges, whereas in three dimensions this forms a mixture of triangular and quadrilateral faces, Fig. 9. Triangular faces are only formed on the boundaries between control cells of differing refinement level. The quadrilateral faces within control cells can be divided into two triangles to ensure a fully triangular mesh.

To ensure the surface is smooth and conforms as well as possible to the geometry contained volume fractions specified by each $v_t$, a volume constrained surface area minimisation is performed. The vertices on this surface mesh are moved locally normal to the surface to minimise the surface area, subject to an equality constraint on contained volume within each control cell. This amounts to solving the constrained optimisation problem given by the Lagrangian in Eq. 16.

16 $$\begin{aligned} {\mathfrak {L}} = A_s + \boldsymbol{\lambda } \cdot \left( \mathbf {v_c} - \mathbf {v_t} \right) \end{aligned}$$

$A_s$ is the surface area, $\mathbf {v_c}$ is a vector of the current geometry contained volume fractions within each control cell, $\mathbf {v_t}$ is the volume-of-solid parameters and $\boldsymbol{\lambda }$ the Lagrange multipliers. This constrained optimisation problem is solved using the sequential quadratic programming (SQP) approach of Boggs and Tolle (1995). This process is efficient as all derivatives of surface area and contained volume fractions with respect to the position of each surface vertex can be evaluated analytically.

## Optimising the behaviour of the cellular automata

To investigate and optimise the behaviour of the cellular automata, it was first applied to a simple test case. Following this, a set of numerical experiments were performed to select optimal values for each of the variable parameters; $R_c$, $R_h$, $N_r$ and $k_v$.

### Investigation and validation of behaviour

To initially investigate the behaviour of the method, a test case involving a single control cell with $v_t = 0.3$ was performed. This involved initialising all sub-grid cell states to ρ=1, and running the scheme to observe its behaviour. This test was performed with $R_c = 32$, $N_r = 4$, $k_v = 0.3$ and $R_h = N_r$, with these values selected after brief numerical experimentation.

**Fig. 10:**
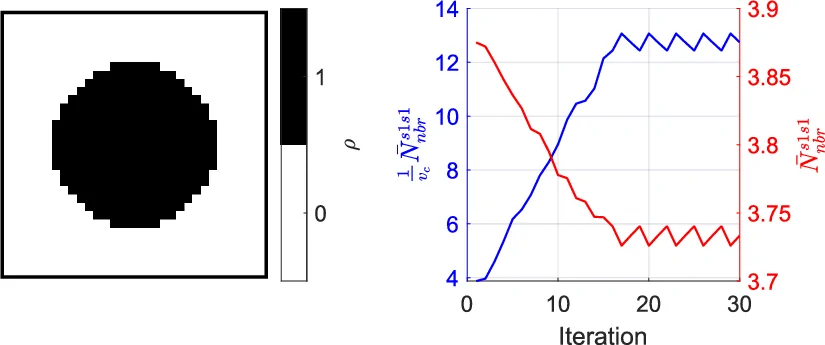
The converged sub-grid cell states and the average number of ρ=1 neighbours of ρ=1 cells, ${\bar{N}}_{nbr}^{s1s1}$

**Fig. 11:**
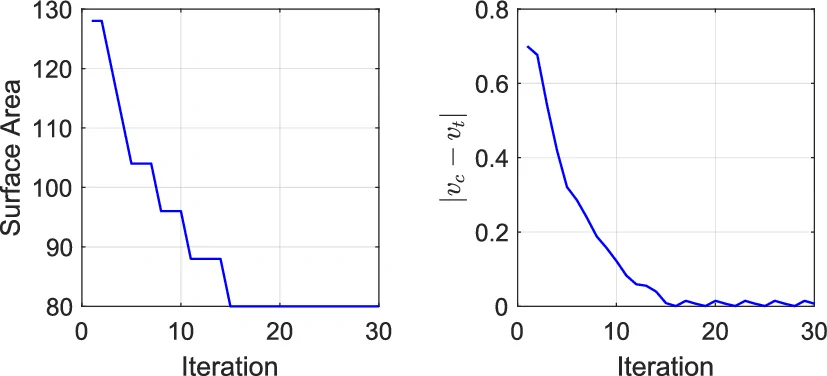
The surface area and volume fraction error in the sub-grid cell states described by the cellular automata during the iteration process

From Figs. 10 and 11, it is possible to confirm that the cellular automata behaves as expected. The average number of ρ=1 neighbours of ρ=1 cells ${\bar{N}}_{nbr}^{s1s1}$ increases as the iteration proceeds, when normalised by the current volume fraction. This confirms that the cellular automata displays behaviour similar to what is shown in Fig. 6. Furthermore, the surface area reduces until converging onto a fixed value, while the volume fraction error is reduced almost to zero. The final distribution of ρ=1 cells also appears similar to what one would expect, a circle, as this shape has the minimum surface area for a given volume.

Looking at Fig. 11, it appears the volume fraction error $d_v$ never settles to either zero or a non-zero fixed value. This is due to the cellular automata settling into a pseudo-steady state condition, where the cell states jump between a few configurations with equal surface areas but differing volumes. This can be explained with the help of Eqs. 7 & 17.

Given the parameters used for this test case with $v_t = 0.3$, the minimum possible volume fraction error is $\min |v_c - v_t| \approx 1.95 \cdot 10^{-4}$ (Eq. 17), thus the target volume fraction $v_t$ cannot be approximated exactly by any number of ρ=1 cells. Hence, the scheme will alternately loop through a set of configurations that activate rule one then rule two in Eq. 7, attempting to find but not achieving an exact match to the target volume fraction $v_t$. This behaviour appears unavoidable, but results in no functional issues for the performance of the scheme.

17 $$\begin{aligned} \min |v_c - v_t| = \left| \frac{\text {nint}(v_t R_c^n)}{R_c^{n}} - v_t \right| \end{aligned}$$

### Selection of variable parameters

There are a set of four constants for which a value must be selected; $R_c$, $R_h$, $N_r$ and $k_v$. These define properties of the sub-grid and the cellular automata scheme itself. $R_c$, $R_h$ and $N_r$ have some inter-dependence between them, whereas $k_v$ is independent of the other three. To select optimal values for each variable parameter, a set of numerical experiments have been performed. These involved varying some of these parameters, and observing the resulting effects on the performance and behaviour of the cellular automata scheme.

These experiments have each been performed using a fixed set of values for each constant that was not varied, each of which was selected following brief empirical experimentation. These are outlined in each relevant section. For each experiment, a target volume fraction of $v_t = \frac{\pi }{6} \approx 0.5236$ was used in all control cells. This was because $\frac{\pi }{6}$ is an irrational number, such that the cellular automata cannot exactly recover this volume fraction with any combination of optional input parameters.

Additionally, when observing the performance of the cellular automata, care must be taken when observing the volume fraction recovery errors at its ‘converged’ state, as it will not always reach a completely steady state, as seen in Fig. 11b. As a result, all measured volume fraction errors for the test cases presented in this section were averaged over 10 iterations once a steady or pseudo-steady state had been reached.

#### Selection of $R_c$ and $N_r$

Due to the interdependence between these parameters, as they together determine $v_{min}$, selection of both was performed together. First, a rule for the selection of $N_r$ was established by performing a sweep of its value in powers of two at both $R_c = 32$ and $R_c = 128$ for a test case using a control mesh of 3×1 cells. Subsequently, a sweep of $R_c$ was performed with the optimal $N_r$ to select an appropriate value of $R_c$. Both of these tests were performed with $k_v = 0.75$ and $R_h = N_r$. Ideally, given the definition of $v_{min}$ Eq. 11, $N_r$ should be set to be as small as possible to maximise the range of possible volume fractions that can be represented by each control cell. However, as $N_r$ governs the maximum range of direct influence between two cells, changing $N_r$ changes the geometry represented by the cellular automata at its converged condition.

**Fig. 12:**
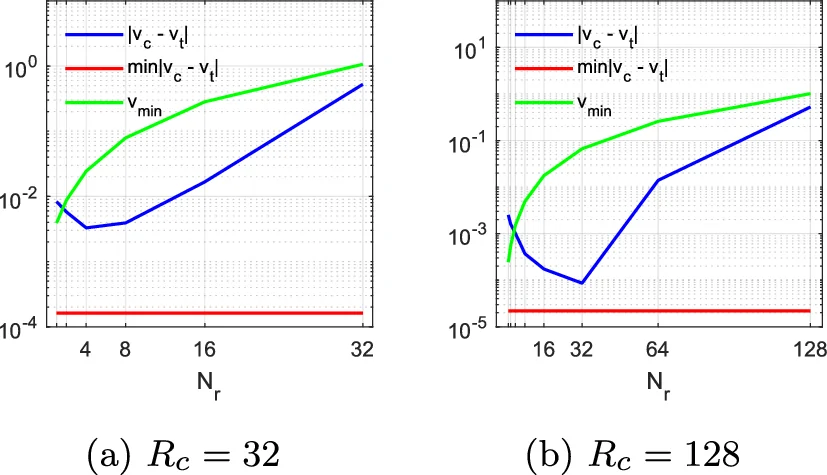
Recovered volume fraction error and $v_{min}$ at varying values of $N_r$ for two values of $R_c$

**Fig. 13:**
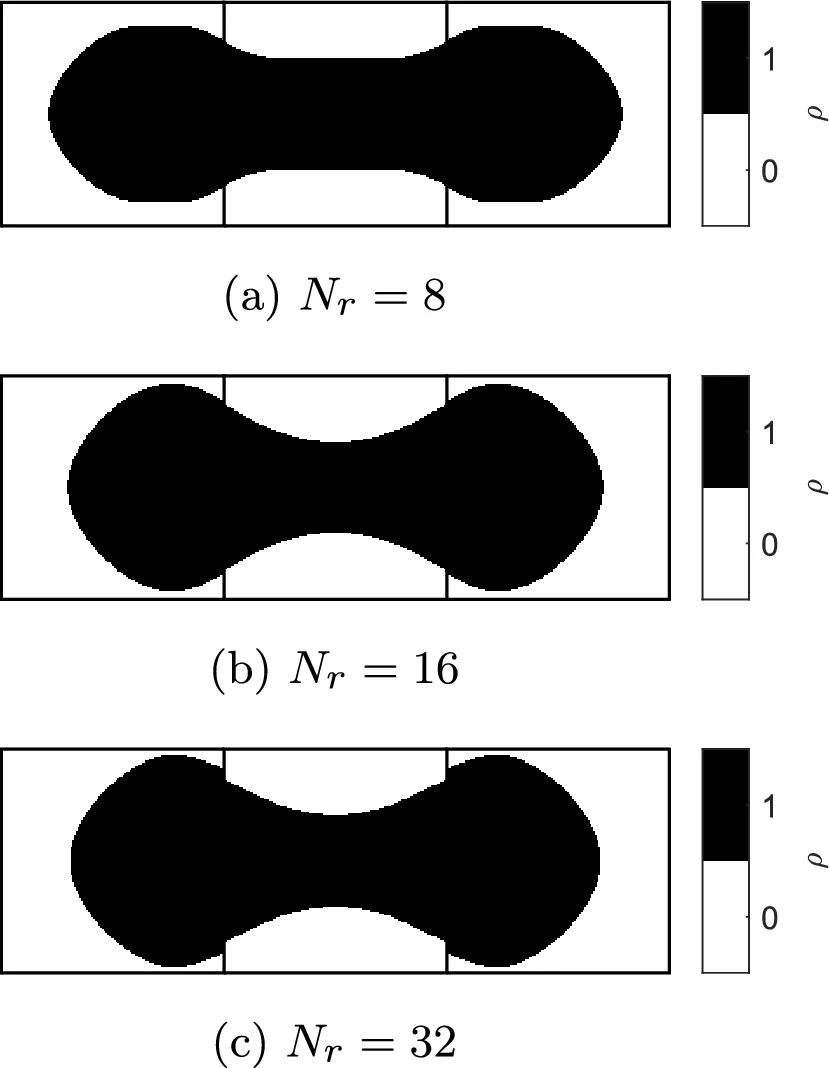
Geometries at varying $N_r$ for $R_c = 128$

From Fig. 12, it is evident that in terms of volume fraction recovery, the best performance for $R_c = 32$ is seen at $N_r = 4$, and for $R_c = 128$ is seen at $N_r = 32$. Looking however at Fig. 13, at $N_r = 128$, discontinuities in the surface position appear at the control cell boundaries using this $N_r$. However, these are eliminated at $R_c = 128$ with $N_r = 16$, with only a minimal impact on volume fraction recovery performance. A similar effect is also seen at $R_c = 32$ with small discontinuities of more than one cell at $N_r = 8$ but none at $N_r = 4$. Furthermore, at both $R_c = 32$ and $R_c = 128$, with $N_r = 4$ and $N_r = 16$ , respectively, the recovered geometry appears most similar to those constructed in a similar case by Payot et al. (2017).

In both cases, these optimal values of $N_r$ are exactly $\frac{R_c}{8}$. Thus, empirically it appears that as the optimal compromise for any $R_c$, selecting $N_r$ through Eq. 18 will likely provide the ‘best’ smooth geometries, smallest volume fraction recovery errors, and a reasonably small $v_{min}$, with $v_{min} = 0.0244$ at $R_c = 32$ and $v_{min} = 0.0176$ at $R_c = 128$.

18 $$\begin{aligned} N_r = \max \left( 1, \frac{R_c}{8} \right) \end{aligned}$$

**Fig. 14:**
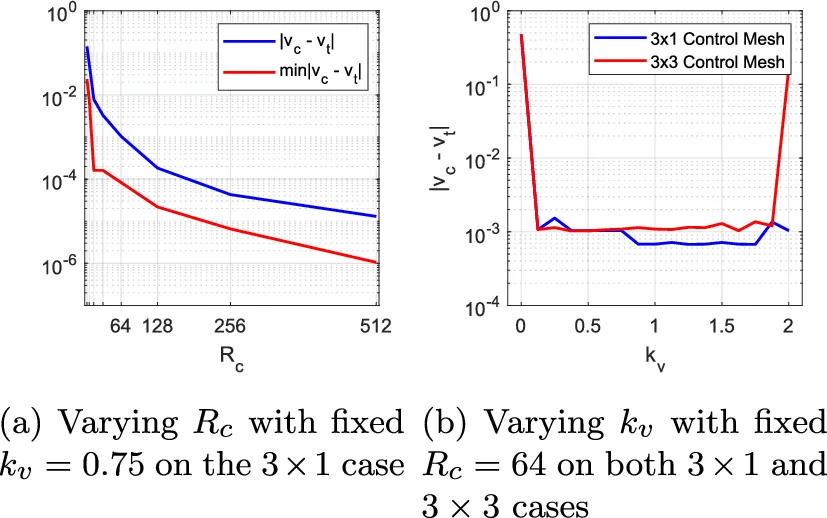
Volume fraction errors with varying $R_c$ and $k_v$

The value chosen for $R_c$ is somewhat less important. Larger values will reduce the theoretical minimum volume fraction error through Eq. 17, however will cause the scheme to become more computationally expensive as the number of sub-grid cells increases. Using the same 3×1 control mesh case, now with $N_r$ selected according to Eq. 18, a sweep of $R_c$ in powers of two from 4 to 512 was performed, with the minimum theoretical and achieved volume fraction recovery errors shown in Fig. 14a.

**Fig. 15:**
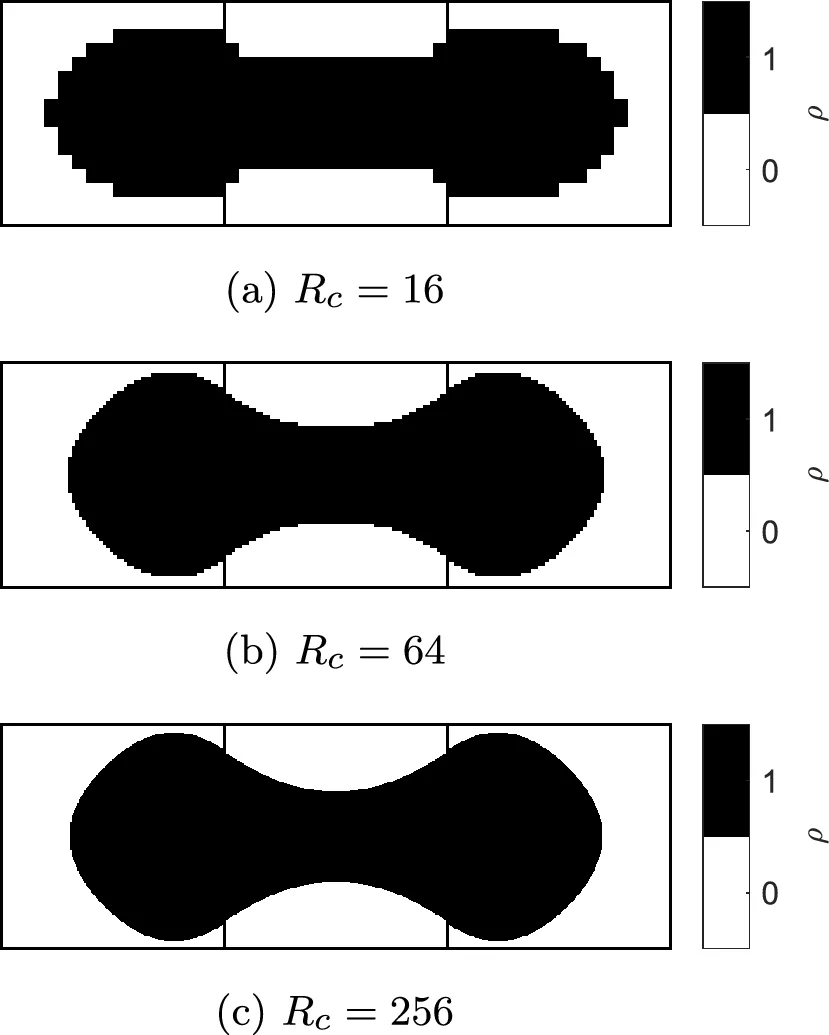
Geometries at varying $R_c$ with the optimal $N_r$

As expected as $R_c$ is increased, both the actual and theoretical minimum volume fraction error reduce. However, for values of $R_c \ge 64$, negligible change in the shape of the geometry was observed, Fig. 15. As a result, choosing $R_c = 64$ as a default value appears a good compromise between volume fraction recovery and computational expense. This is the smallest value for $R_c$ at which increasing $R_c$ does not significantly change the geometry constructed by the scheme, with a $v_{min} = 0.0198$ and a volume fraction resolution of $v_{res} = 2.44 \cdot 10^{-4}$. In practice, any value for $R_c \ge 32$ will likely perform well; however, for most cases, $R_c = 64$ appears likely the optimal choice.

#### Selection of $k_v$

The chosen value of $k_v$ affects how strongly the error in the contained volume fraction influences the value of $h_b$ though Eq. 10. To select a good value for $k_v$, two test cases were investigated, one using a 3×1 control mesh and the other a 3×3 control mesh. These again used $v_t = \frac{\pi }{6} \approx 0.5236$ in all control cells. The 3×3 case is important in this instance as during the solution process all three of the main rules in Eq. 7 are activated as the parameterisation constructs an internal void in the central control cell.

During empirical experimentation, it was found that values of $k_v \approx 1$ provided the optimal behaviour. As such, a sweep in values of $k_v$ around 1 was performed on both the 3×1 and 3×3 control mesh cases.

Looking at Fig. 14b, it is clear that for both cases for $0.25 \le k_v \le 1.75$ the recovered volume fraction error is relatively insensitive to the value of $k_v$. For the 3×3 case, however for $k_v$ outside of this range the internal void was not correctly constructed, leading to a large volume fraction error. For $k_v < 0.25,$ all sub-grid cells in the central control cell remained stuck at ρ=0, whereas for $k_v> 1.75$ the same sub-grid cells remained stuck at ρ=1. For both cases however, for all values of $k_v \in [0.25,1.75],$ the geometry appears insensitive to the value of $k_v$, with it in each case tested being almost identical.

Consequently, it appears that any value of $k_v \in [0.25,1.75]$ will likely perform well; however, the region of $0.5 \le k_v \le 0.75$ is where both cases perform most similarly. As such somewhere in this range is likely a good choice for a default value. Hence, as a larger value of $k_v$ will in theory lead to a more rapid reduction in the geometry contained volume fraction errors in each control cell, a value of $k_v = 0.75$ was chosen as the default optimum value, as this is the largest value for which both cases performed the most similarly.

#### Selection of $R_h$

The value chosen for $R_h$ has no effect on the performance of the scheme, but will affect the format that control meshes can take through Eq. 2. As a result, it is most simple to assign this a fixed value that is as small as possible for the chosen combination of control mesh, $R_c$ and $N_r$. $R_h$ must be bounded only by $N_r \le R_h \le R_c$, thus in most cases it can be set though Eq. 19. This ensures a suitable value for $R_h$ is selected that is at least as large as $N_r$ and ensures the limits imposed though Eq. 2 are adhered to between all adjacent control cells $C_i$ and $C_j$ within the control mesh.

If the value of $R_h$ returned by Eq. 19 is larger than $R_c$, then the control mesh contains a jump in refinement level $\Delta L_r$ between two adjacent control cells $C_i$ and $C_j$ that is too large for the chosen values of $R_c$ and $N_r$. This then necessitates an increase in $R_c$ and correspondingly in $N_r$ to be run without modification to the control mesh.

19 $$\begin{aligned} R_h = \max {\left\{ \begin{array}{ll} 2 \\ N_r \\ \max _{C_i / C_j} \left( \frac{1}{2^{\Delta L_r}} \right) \\ \max _{C_i / C_j} \left( 2^{\Delta L_r} \right) \\ \end{array}\right. } \end{aligned}$$

#### Summary of optimal parameters

Table 1 summarises the optimal variable parameters chosen for the sub-grid and cellular automata scheme in this section. These values are used throughout the remainder of this work.

**Table 1:** Optimal sub-grid and cellular automata variable constants, and properties of the control mesh deriving from their choices

$R_c$	$R_h$	$N_r$	$\max \left( \Delta L_r \right)$ at $R_h = 8$	$k_v$
64	≥8	8	3	0.75

## Response of the surface to changes in $v_t$

Evaluation of the sensitivity of the smooth parameterised surface position to changes in the volume-of-solid parameters $v_t$ is essential for the application of this parameterisation within a gradient-based optimisation procedure. As the cellular automata is an integer process, it is not differentiable. If a finite difference approximation is instead used through the cellular automata, as its converged state does not change smoothly with $v_t$, this would likely yield a noisy approximation to the surface sensitivity to changes in each $v_t$. Therefore, to estimate the surface sensitivities to changes in each $v_t$, the sensitivity of the smoothing algorithm alone was utilised. Assuming the smoothing procedure can always recover the target contained volume fractions to within some small tolerance ϵ, even with non-smooth changes in the solution of the cellular automata, the changes in surface position post-smoothing should be smooth and differentiable.

Given the smoothed surface is the result of an equality constrained SQP optimisation, the method of Büskens and Maurer (2000) can in theory be used to evaluate the surface sensitivities analytically. It was found in practice however, that this method did not well capture the surface response outside of each control cell. As such a finite difference approximation was used. This procedure requires first evaluating the cellular automata result and applying the smoothing algorithm. Subsequently, starting from the smoothed surface, each parameter $v_t$ is perturbed in turn, and the smoothing procedure run again in the sub-set of control cells forming the 2-ring neighbourhood around the control cell for which $v_t$ was perturbed. The sensitivity of the surface to changes in each $v_t$ can then be evaluated by comparing the positions of each surface vertex between the initial and perturbed surfaces, using standard finite difference approximations.

**Fig. 16:**
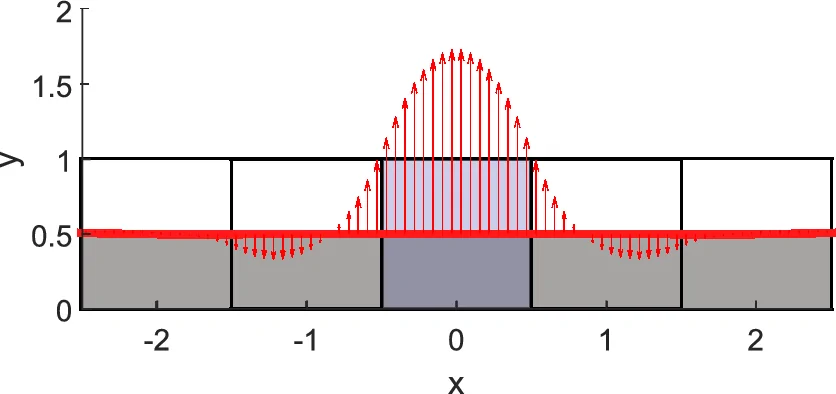
Surface sensitivity to changes in $v_t$ in the blue highlighted control cell for a section of flat surface. The grey shading represents the sub-grid cell states

**Fig. 17:**
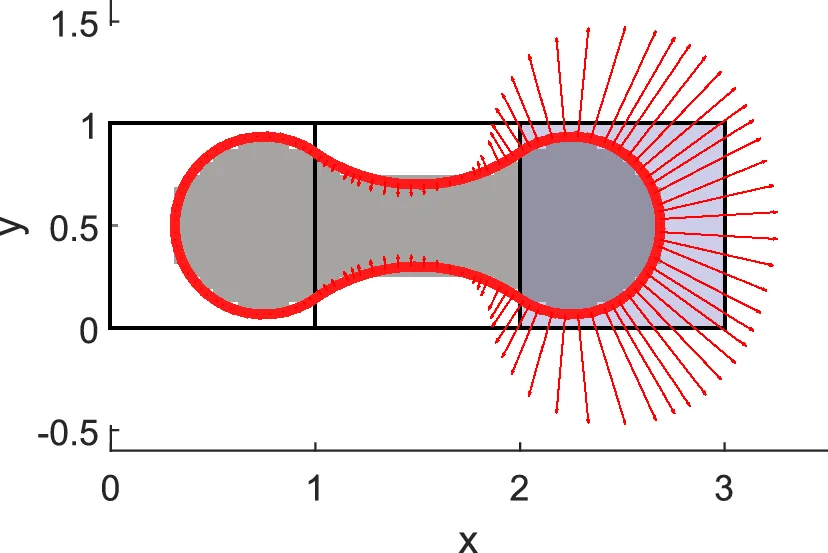
Surface sensitivity to changes in $v_t$ in the blue highlighted control cell for a dumbbell type geometry. The grey shading represents the sub-grid cell states

Figures 16 and 17 show some examples of the surface response to the change of one $v_t$ on different geometries.

This procedure for evaluating the surface sensitives does however have one major disadvantage. This is that it is not possible to evaluate the surface response to changes in $v_t$ for control cells adjacent to but not containing the surface. This can be an issue as it does not allow the evaluation of sensitivities to changes to the ‘active set’ of control cells, those that contain the surface with $0< v_t < 1$. When this parameterisation is applied within a gradient based optimisation this can be a disadvantage. Evaluation of sensitivities to changes in the active set of control cells can only be evaluated with a finite difference through the entire scheme, the cellular automata and surface smoothing; however, this is very computationally inefficient.

## Geometric reconstruction

The performance of a parameterisation when used to re-construct existing geometries can provide insights into its capabilities. To re-construct existing geometry using a volume-of-solid method such as the one presented in this work, the contained volume fractions $v_t$ of the target geometry when overlaid upon an existing control mesh must be evaluated. This can be done using a polygon clipping algorithm, such as the method of Sutherland and Hodgman (1974). This is used to clip the target geometry to each control cell in turn, then find each $v_t$ as the ratio of the volume of this clipped geometry to that of its containing control cell. In this section, this process has been used to re-construct the aerofoils within the UIUC aerofoil library, Masters et al. (2016). The errors in the re-constructed geometries were then compared against the wind tunnel tolerance (WTT) specified by Kulfan and Bussoletti (2006).

**Fig. 18:**
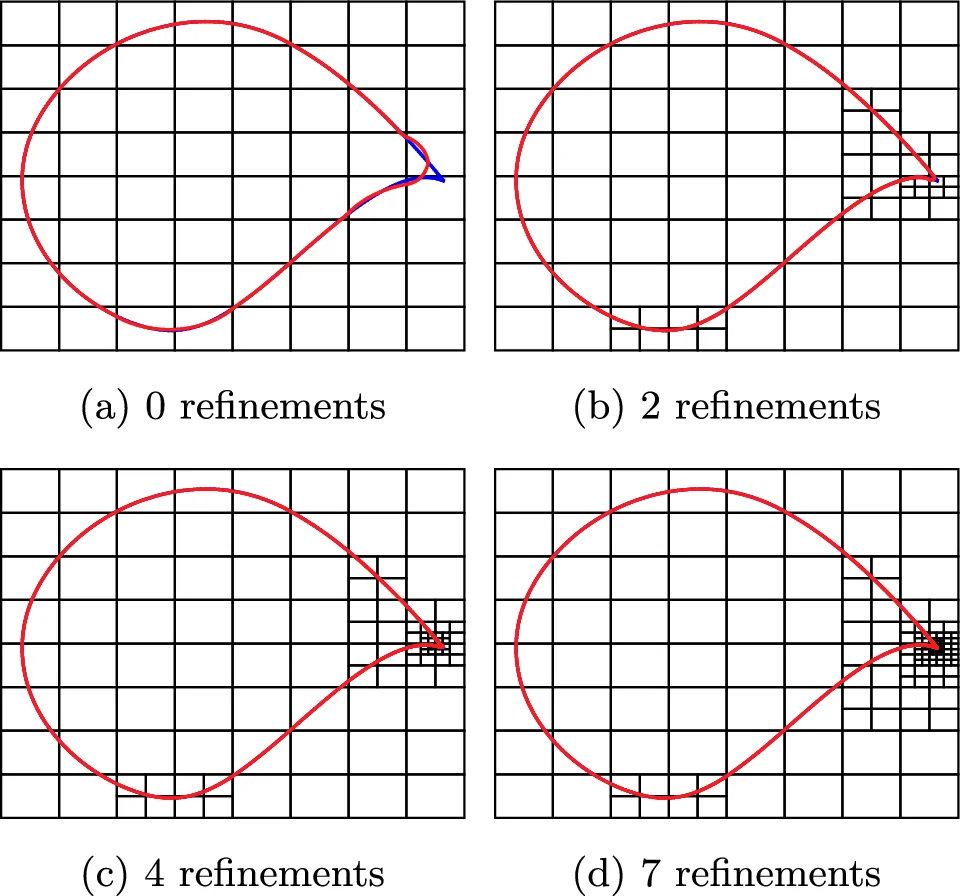
The reconstruction (red) of an RAE2822 aerofoil (blue) using an initially 8×8 control mesh refined using the metric defined in Eq. 20

To automatically and accurately re-construct geometries using this clipping procedure, refinement of the control mesh can be applied recursively in regions where the geometric error between the target and re-constructed geometries is greatest. To do this, starting from a base control mesh, the target geometry was clipped and re-constructed on this control mesh, the error *E* in the normal direction between the target and re-constructed geometries evaluated, then the control cells *C* tagged by the refinement metric $M_r$ defined in Eq. 20 were refined. This process was repeated recursively from a base 8×8 control mesh up to a maximum of 7 times, Fig. 18.

20 $$\begin{aligned} M_r(E) = {\left\{ \begin{array}{ll} \text {yes, if: } \max (E \in C) \ge 10^{-4}\\ \text {no, else} \end{array}\right. } \end{aligned}$$

**Fig. 19:**
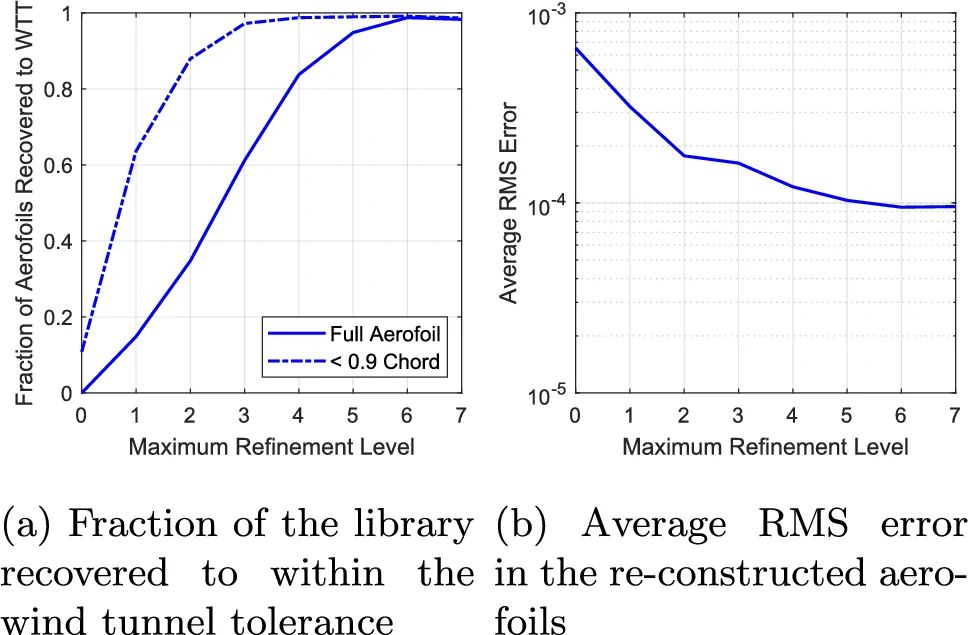
The performance of the parameterisation at approximating the geometries within the UIUC aerofoil library

**Fig. 20:**
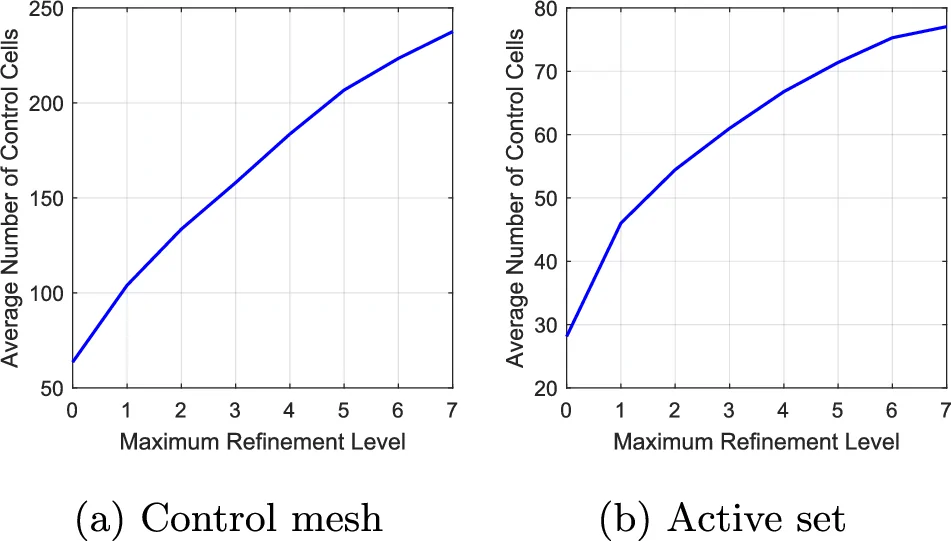
The average number of control cells in the mesh, and active set defining the surface of the geometry, with different maximum control mesh refinement levels

The results of applying this procedure to all geometries within the UIUC aerofoil library are summarised in Fig. 19 and 20.

**Fig. 21:**
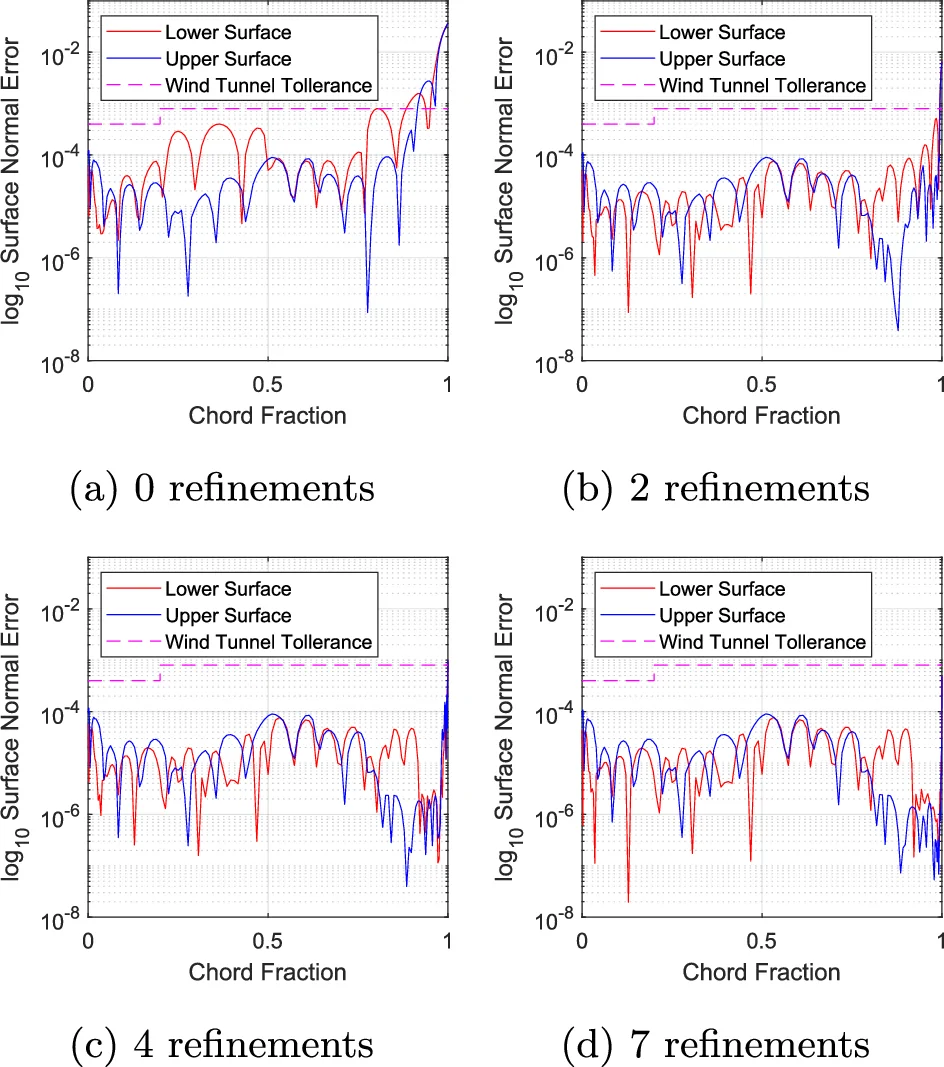
The distribution of geometric errors in the re-construction of an RAE2822 aerofoil with different maximum control mesh refinement levels

As shown by Fig. 19a, with enough permitted control mesh refinement the parameterisation was able to re-construct 98.3% of the aerofoils within the library to within the wind tunnel tolerance. In most cases, until higher maximum control mesh refinement levels were permitted, the geometric errors were concentrated around the sharp rear sections of aerofoils, Fig. 21. Consequently, ignoring this region of the aerofoils, far fewer refinements were required to re-construct similar fractions of the aerofoil library to an equivalent tolerance.

Additionally, the average root mean squared (RMS) error between the re-constructed and target geometries plateaued at approximately $10^{-4}$, even when further refinement was permitted, fig. 19b. This was due to the control cell error refinement tolerance set within Eq. 20, as when the maximum error at any point reached this value or smaller, the control cell would not be refined further, and the average geometric error in the re-constructed geometry would not reduce. If this error bound in Eq. 20 were set to a smaller value, it is likely the average RMS error would reduce further with increases in permitted refinement level.

However, it is worth noting that as shown by Fig. 20, the average number of design variables that define the surface geometry (the ‘active set’ of control cells that contains the geometry surface with $0< v_t < 1$) of the aerofoils becomes relatively large, of the order of 75 when up to 7 levels of control mesh refinement were permitted. This is significantly greater than the approximately 20→30 design variables that many existing parameterisations require for similar levels of geometric recovery of the same aerofoil library, Masters et al. (2016).

High dimensionality is often a problem for aerodynamic shape optimisation, reducing the rate of convergence and increasing the likelihood of the optimisation becoming stuck in a local minimum. Consequently, at a glance, this may indicate that this parameterisation would perform worse than many excising methods for aerodynamic shape optimisation. However, by exploiting an optimisation framework similar to that investigated by Masters et al. (2017), utilising in this case a sequential set of optimisation then control mesh refinement cycles, similar to what was done in this section, the number of design variables can be significantly reduced until the geometry is already close to optimal. As shown by Masters et al. (2017), this method improves the performance of high-dimensional optimisation, and thus when applied using this parameterisation should avoid many of the issues that may be caused by its potential for high dimensionality.

## Shape and topology optimisation

To evaluate the capabilities of the presented CAVoS parameterisation, it has been applied to a set of two-dimensional aerodynamic shape optimisation cases. The first set of cases involved fixed topology gradient-based optimisation. To benchmark the performance of the parameterisation, the aerodynamic performance of the optimised geometries resulting from these cases has been compared to existing analytically derived optimal geometries.

Subsequently, a set of gradient-free topology-inclusive optimisation cases were attempted. These have been used to demonstrate the ability of the parameterisation to explore geometries of varying topology within a single optimisation. The final case of this type presented is identical in objective and constraints to one of the fixed topology gradient-based cases, and the result is thus used to highlight the potential improvements in aerodynamic performance that are possible by exploiting topology-inclusive shape optimisation.

### Optimisation methodology

Aerodynamic shape optimisation requires the coupling of the geometry parameterisation to the tools required to evaluate the aerodynamic performance of the geometry, and an optimiser to apply changes to the geometry to improve its performance for some objective. For the cases presented in this section, the geometry was represented using the cellular automata-based CAVoS method presented in this work. The parameterisation was configured using the parameters described in Table 1. To construct and smooth the surface geometry, the converged sub-grid cell states in each control cell were re-sampled down to $R_c = 16$ to coarsen the surface mesh. The design variables to control the geometry were the volume-of-solid parameters $v_t \in [0,1]$ in each control cell.

Evaluation of the flow field around each geometry and the resulting force coefficients was performed with an inviscid, compressible Euler solver using the Jameson cell centred approach (Jameson et al. 1981), based on the implementation of the Edge solver by Eliasson (2002). Gradients of the force coefficients with respect to the geometry surface position were evaluated with a discreet adjoint method based on the implementation of Kedward et al. (2020). Volume mesh generation was performed using a Cartesian cut-cell approach, Aftosmis et al. (1998). This method allows robust construction of a volume mesh for arbitrary geometries consisting of an arbitrary number of different objects. This ensures it is possible to aerodynamically evaluate all geometries constructed by the CAVoS parameterisation, even if they contain multiple and possibly nested objects.

### Constrained volume drag minimisation at mach 2

A challenging optimisation case to test the performance of the CAVoS parameterisation is that of the minimisation of the drag coefficient, $C_d$, under a constraint of fixed volume in supersonic flow, Eq. 21. Comparisons can be made to the set of existing analytical results for this case. Sears (1947) and Haack (1941) independently derived optimal three-dimensional geometries for this case using linearised aerodynamic relations, leading to the well-known Sears-Haack body. In two dimensions, as presented by Palaniappan and Jameson (2010), linearised results show the optimum is parabolic, providing the volume of the geometry is small. Klunker and Harder (1952) also used a semi-analytical non-linear method to derive truncated ogive type optimal geometries for larger contained volumes. Previously, this case has also been attempted by Payot et al. (2019a) using numerical optimisation and an R-snakes-based volume-of-solid parameterisation.

21 $$\begin{aligned} \begin{aligned} \text {min }&: C_d \\ \text {subject to }&: V - V_t = 0 \\ \end{aligned} \end{aligned}$$

**Fig. 22:**
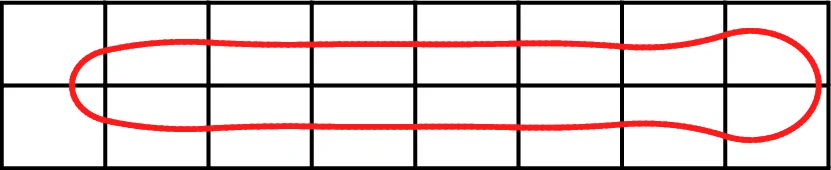
The initial geometry used for the $V_t = 0.1$ case overlaid onto its control grid. This geometry was the same for all cases but scaled in the *y* direction

Here, a set of 6 cases have been run at varying contained volumes $V_t$ from $V_t = 0.025$ to $V_t = 0.150$ at Mach 2. Each case was initialised using a control mesh of 8×2 cells, with overall dimensions of 1 in the *x* direction and $2V_t$ in the *y* direction. The volume-of-solid parameters were initialised to $v_t = 0.5$ in all control cells except the two at minimum *x*, which were instead initialised to $v_t = 0.1$, Fig. 22.

The geometry constructed by the parameterisation at each iteration was scaled such that its chord was exactly one for evaluation of the drag coefficient. A symmetry constraint was applied in the *y* direction to avoid the exploration of non-optimal lifting geometries. Consequently, the optimiser only controlled only half of the volume-of-solid parameters, those at y<0, with the values for y>0 constructed by a reflection of the y<0 values. The optimisation was solved using an equality constrained sequential quadratic programming (SQP) method, Boggs and Tolle (1995), with a BFGS approximation for the objective Hessian, Fletcher (2000).

**Table 2:** Drag coefficients for the 2D sliced Sears-Haack, Parabolic, Klunker-Harder, initial and optimised geometries for each contained volume constraint

**Contained Volume**	$\boldsymbol{0.025}$	$\boldsymbol{0.050}$	$\boldsymbol{0.075}$	$\boldsymbol{0.100}$	$\boldsymbol{0.125}$	$\boldsymbol{0.150}$
**Sears-haack (2D slice)** $\boldsymbol{C_d|_{sh}}$	0.004636	0.018937	0.043741	0.078967	0.118997	0.164408
**Parabolic** $\boldsymbol{C_d|_{pb}}$	0.004334	0.017414	0.039480	0.071009	0.113070	0.168637
**Klunker-harder** $\boldsymbol{C_d|_{kh}}$	N/A	0.017086	0.036715	0.058151	0.084270	0.113353
**Initial** $\boldsymbol{C_d|_{in}}$	0.021766	0.061554	0.102310	0.147601	0.201824	0.257566
**Optimised** $\boldsymbol{C_d|_{op}}$	0.004406	0.016818	0.037081	0.059140	0.087544	0.116509
$\boldsymbol{C_d|_{op} - C_d|_{in}}$ $\boldsymbol{\%}$	-79.76	-72.68	-63.76	-59.93	-56.62	-54.77
$\boldsymbol{C_d|_{op} - C_d|_{pb}}$ $\boldsymbol{\%}$	+1.67	-3.42	-6.08	-16.71	-22.58	-30.91
$\boldsymbol{C_d|_{op} - C_d|_{kh}}$ $\boldsymbol{\%}$	N/A	-1.57	+1.00	+1.70	+3.89	+2.78

As was shown in section 5, the presented CAVoS parameterisation often requires many design variables to accurately represent geometry. This however can be an issue for optimisation, reducing the rate of convergence and increasing the likelihood of locating a local optimum. In previous work by Masters et al. (2017), it has been shown that by using a parameterisation that provides hierarchical levels of geometric control to the optimiser, significant improvements to both the rate of convergence and the objective value achieved upon termination of a high dimensional optimisation can be realised. In this section, localised refinement of the control mesh has been used to implement a similar method using the CAVoS parameterisation. This was done through a sequential set of three optimisation-refinement cycles, with $8 \rightarrow 20 \rightarrow 50$ design variables.

**Fig. 23:**
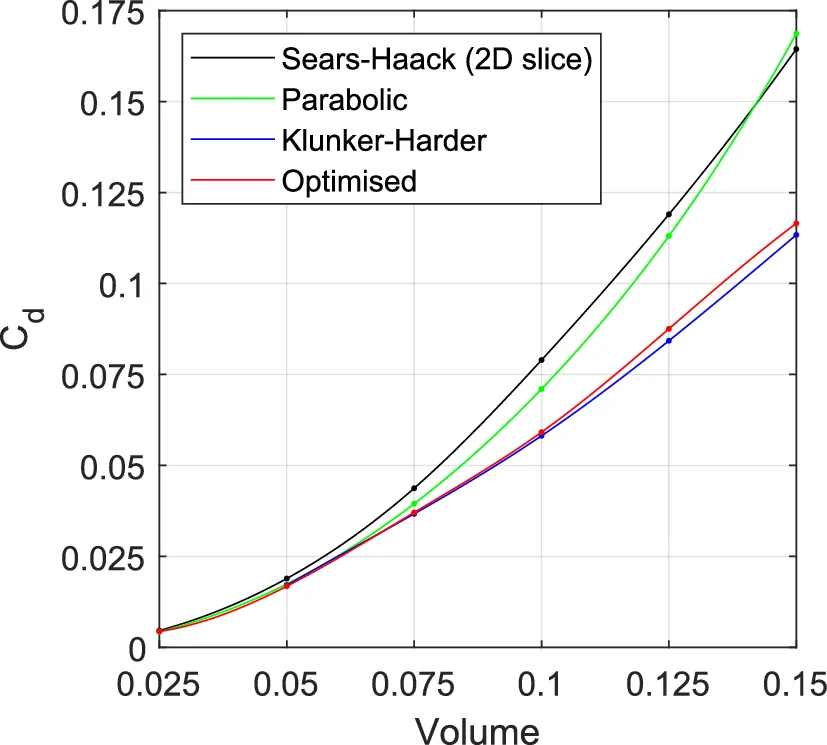
Drag coefficients for the 2D Sears Haack Slice, Parabolic, Klunker-Harder and optimised geometries at varying contained volumes

**Fig. 24:**
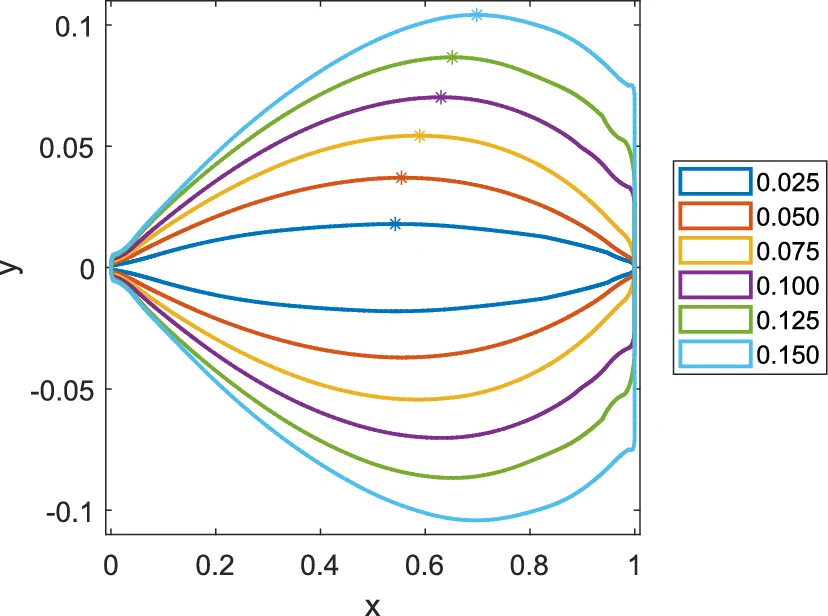
Optimised geometries at varying contained volumes. The point of maximum thickness is marked with an asterisk for each case

Within every cycle, first an optimisation of the geometry on the current control mesh was performed until some minimum step norm tolerance was reached. Here, this was set to $10^{-5}$. Subsequently, upon this termination condition being reached, the control cells in regions surrounding the front and rear sections of the geometry were refined by one level. The geometry was then approximately re-constructed on the refined control mesh, using the polygon clipping algorithm of Sutherland and Hodgman (1974) to evaluate the required contained volume fractions $v_t$ on the new refined control mesh. The results of these optimisations are summarised in Table 2 and Fig. 23, with the optimal geometries for each case shown in Fig. 24.

**Fig. 25:**
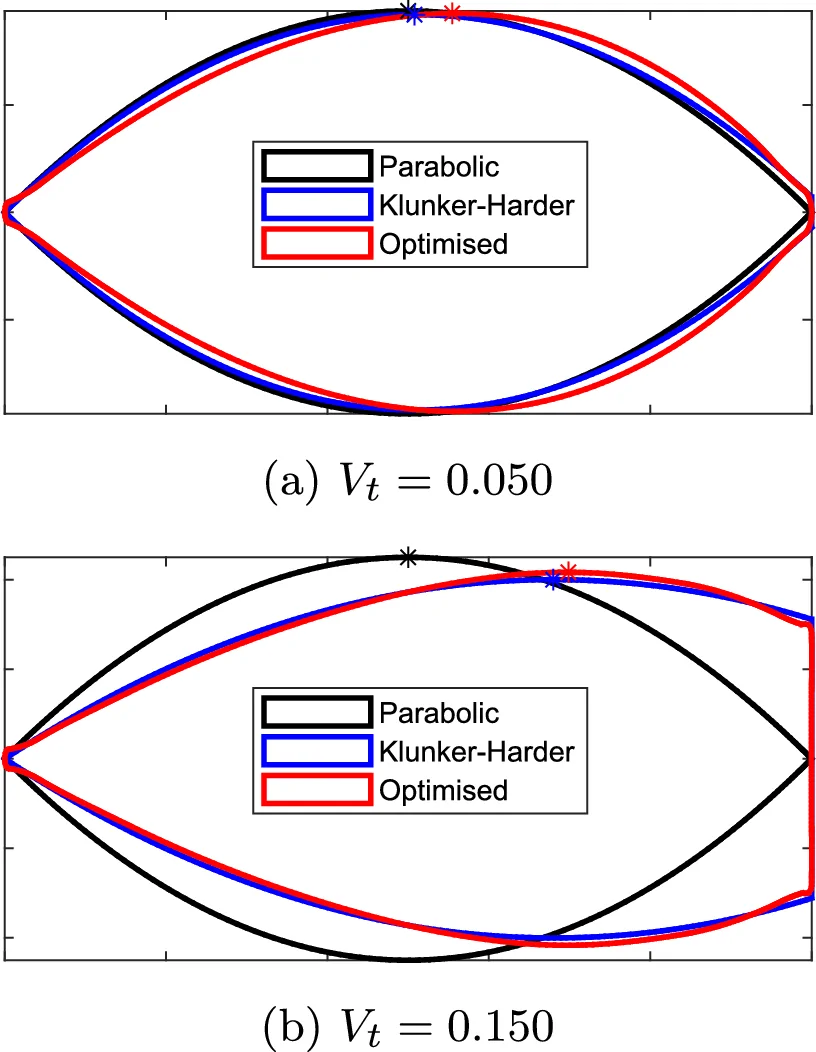
Comparison of the optimised to analytical geometries at two contained volumes. The point of maximum thickness is marked with an asterisk for each geometry

The optimised geometries appear to show the expected trend, with small volumes constructing geometry similar to the linear optimal parabolic ogives, albeit with a blunter rear section, whereas the larger volumes resulted in geometries very similar to the Klunker-Harder ogives, Fig. 25. This occurs as there is a trade off when shifting volume towards the rear of the geometry; between having a shallower angle at the front, resulting in a weaker shock, but having a steeper contraction at the rear, leading to a lower back pressure and more pressure drag. As the volume increases, it becomes more beneficial to weaken the shock at the front of the geometry as the drag caused by this is significantly greater than the pressure drag gained by constructing a blunt rear section.

Looking at Fig. 25, it can be seen in both cases of small and large volume that the optimal geometry has a shallower front section angle compared to even the Klunker-Harder geometry, thus highlighting the importance of weakening this shock. Although it is notable that in both cases the parameterisation has not been able to construct a truly sharp front to each geometry. However, with additional control mesh refinement, the front of the geometry would approach a truly sharp point.

Looking at both Table 2 and Fig. 23, one can observe the optimal geometries have improved on the parabolic geometries and the two dimensional slice of a Sears-Haack geometry for most contained volumes, but only improved on the Klunker-Harder geometry at a volume constraint of $V_t = 0.05$. This is likely a result of the parameterisations inability to construct a truly sharp front section, which becomes more significant with the increasing volume constraint as the *y* direction resolution of the sub-grid mesh reduces. Applying further control mesh refinement around the front section of the geometry would however likely alleviate this issue, providing further reductions in $C_d$.

However, to allow robust optimisation with further control mesh refinement, an approximation for the sensitivity of the surface position to changes in $v_t$ in control cells near to but not containing the surface must be found. This would allow the active set of control cells to change during the optimisation, preventing the structure of the control mesh artificially constraining the geometry.

**Fig. 26:**
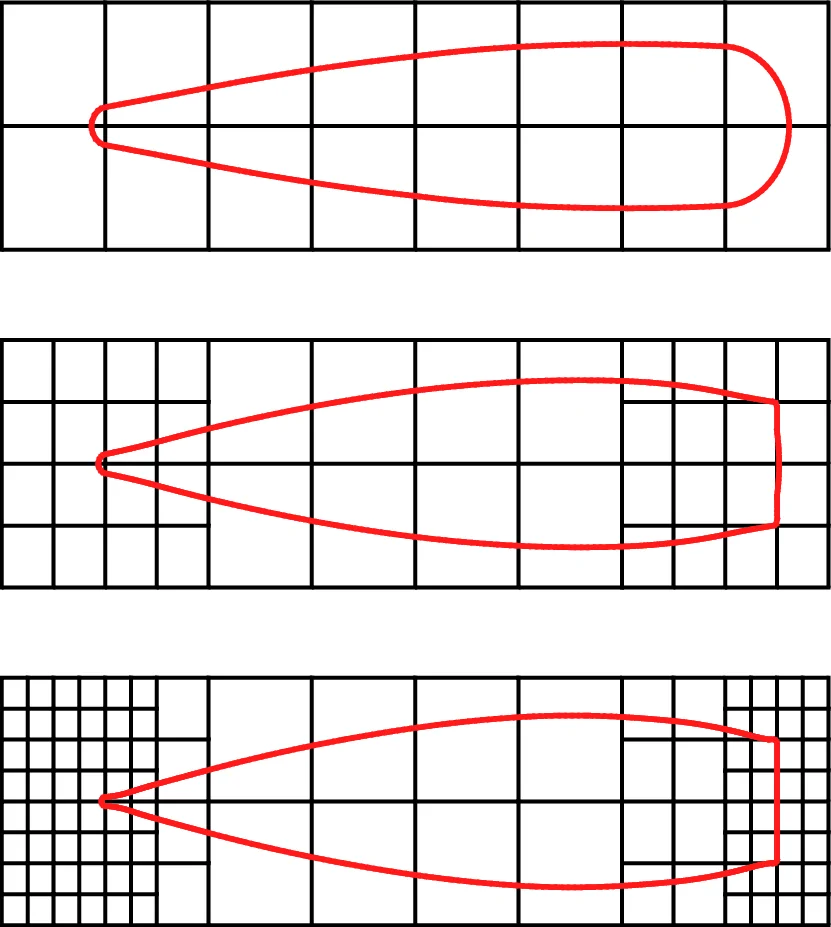
Optimum geometry for the case of $V_t = 0.150$ constructed by the cellular automata after each of the three optimisation-refinement cycles. In each case, it is shown overlaid onto the control mesh used for its cycle

This would likely provide further improvements to the largest two volume cases presented here. In both cases, non-smooth regions of geometry near the rear section corner have been constructed by the surface coming up against the edge of a control cell. As seen in Fig. 26, the case of $A_t = 0.150$ would likely especially benefit from the ability to change the active set of control cells. This would allow the blunt rear section to grow in the *y* direction while maintaining a sharp corner, thus shifting more volume rearwards and more closely approximating the better performing Klunker-Harder geometry.

### Aerodynamic packaging at mach 2

To demonstrate the capabilities of the presented parameterisation to explore differing topologies, a set of optimisation cases have been performed involving the aerodynamic packaging of some existing geometry with the objective of minimising the drag coefficient in Mach 2 flow. Three cases have been attempted, each considering the packaging of two rectangular objects with different distances of 0.05, 0.1 and 0.3 between them. In each case, the pair of objects was defined using two control cells of fixed $v_t = 1$. In each case, the optimiser was permitted control over the $v_t$ in all control cells that did not define the two objects, thus ensuring only addition to the base geometry. Additionally, a symmetry constraint was applied in the *y* direction by reflecting the volume-of-solid parameters across y=0 to avoid the exploration of non-optimal lifting geometries. As such, the optimisation problem was defined by Eq. 22.

22 $$\begin{aligned} \begin{aligned} \text {min }&: C_d \\ \text {subject to }&: v_{t\text { objects}} = 1 \\&: v_t(y> 0) = v_t(y < 0) \\ \end{aligned} \end{aligned}$$

However, due to the method by which the objects were defined, and the simplicity of enforcing the *y*-direction symmetry constraint separately to the optimiser, the optimisation problem itself was unconstrained. As such, to allow exploration of different topologies by avoiding potential local minima, the optimisation was solved using a gradient-free differential evolution optimiser, Storn and Price (1997). This is an agent-based optimisation algorithm, where at each iteration the position in the design space of each agent is updated on a greedy condition as a function of the positions of other agents within the population. For each case, the optimiser was configured as recommended by Storn and Price (1997), with the number of agents in the population approximately 10× the number of design variables. Each optimisation was run until negligible change in the population average objective value was observed.

**Fig. 27:**
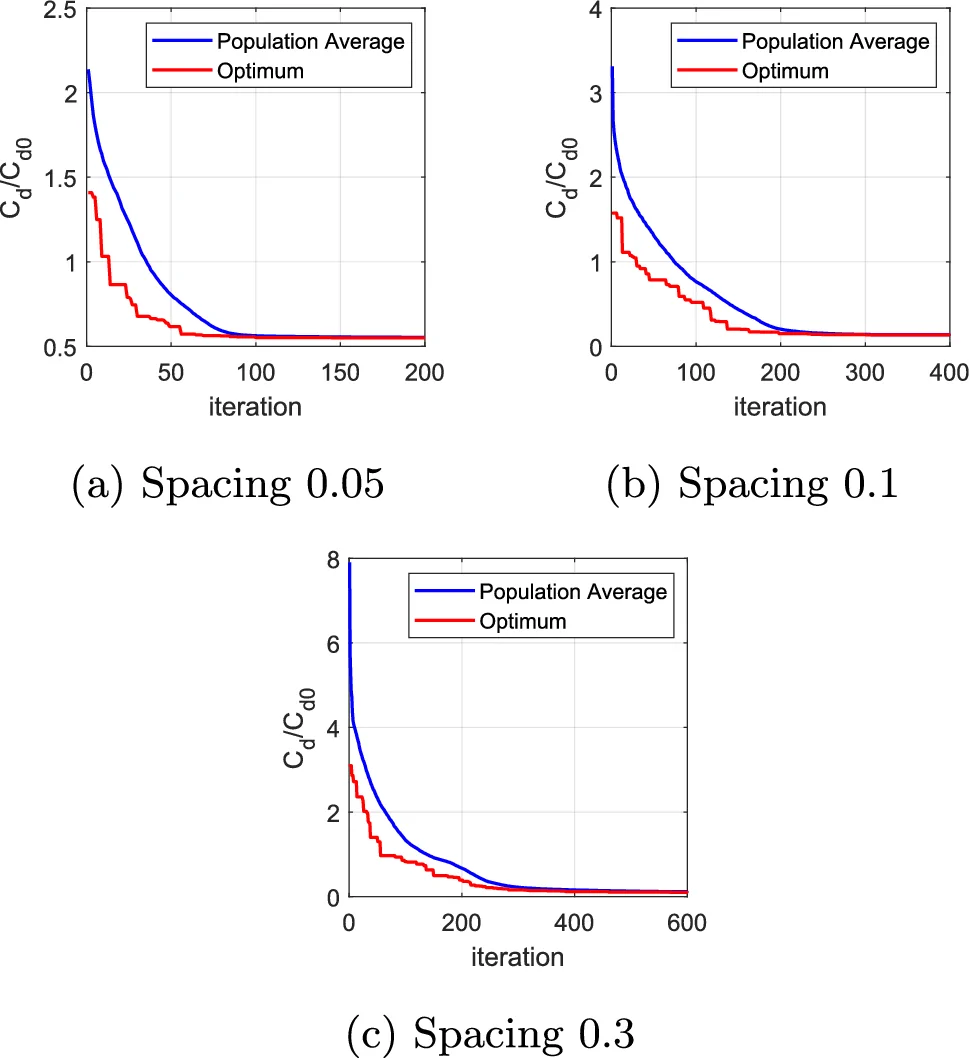
The convergence history of each differential evolution optimisation

**Fig. 28:**
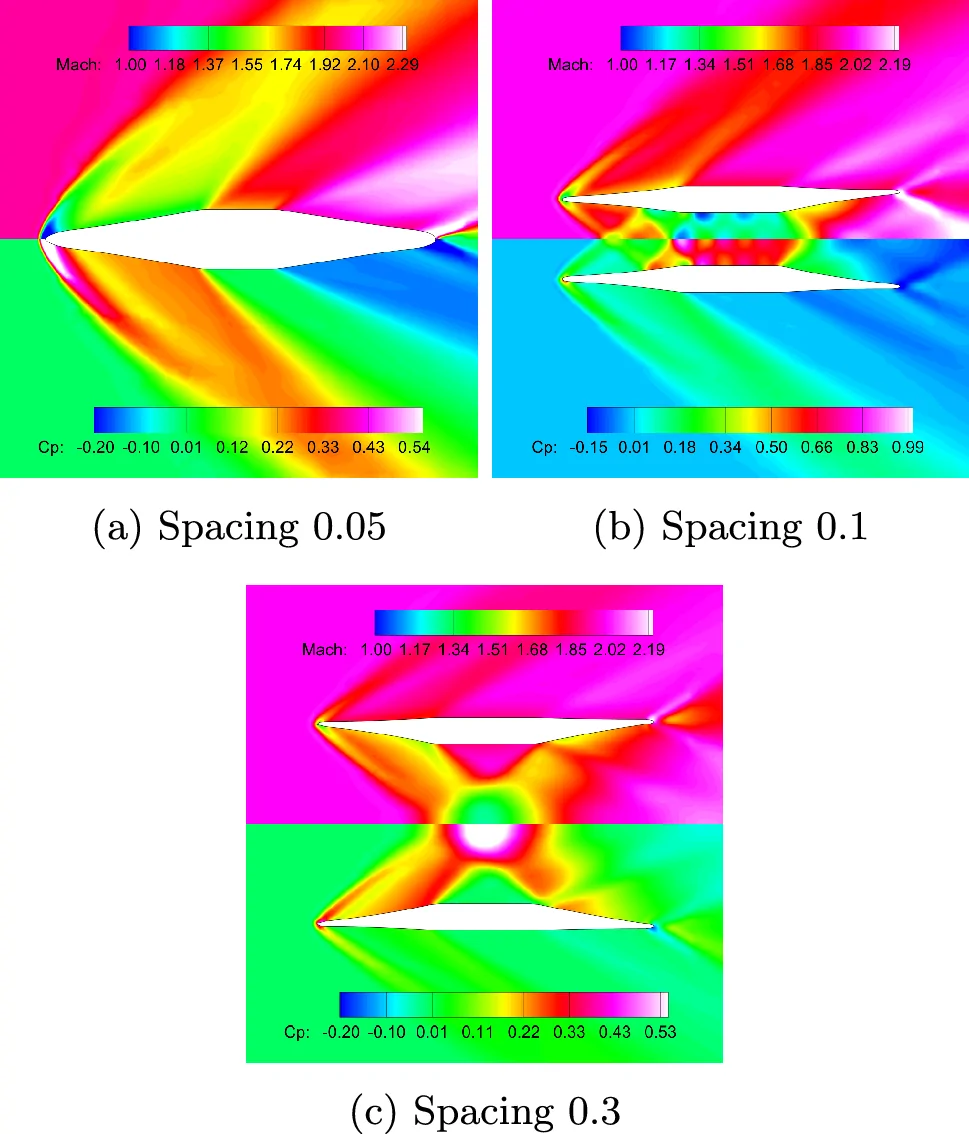
Mach and pressure coefficient fields for the optimal packaged geometry for each case of differing spacing. Each plot is not at equivalent scale

**Fig. 29:**
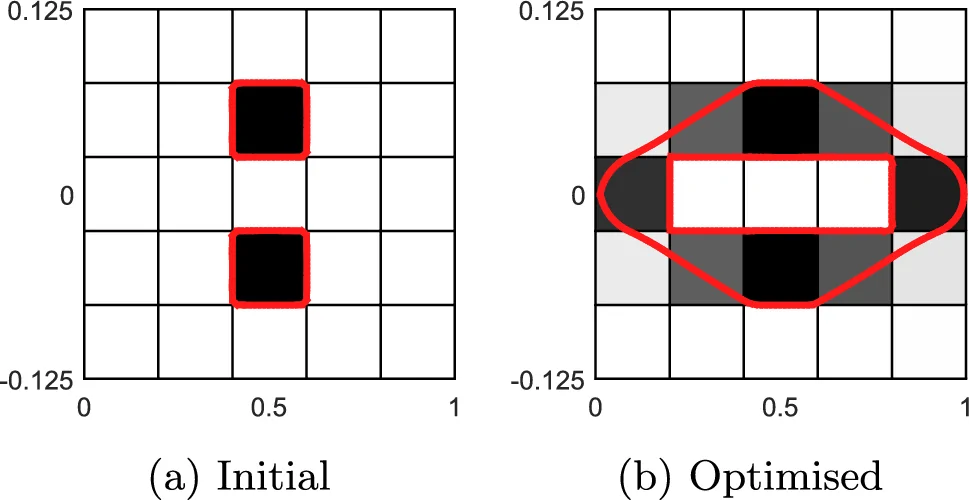
Initial and optimised geometry (red) for the 0.05 spaced case overlaid onto its control mesh. The shading represents $v_t$ in each control cell

**Fig. 30:**
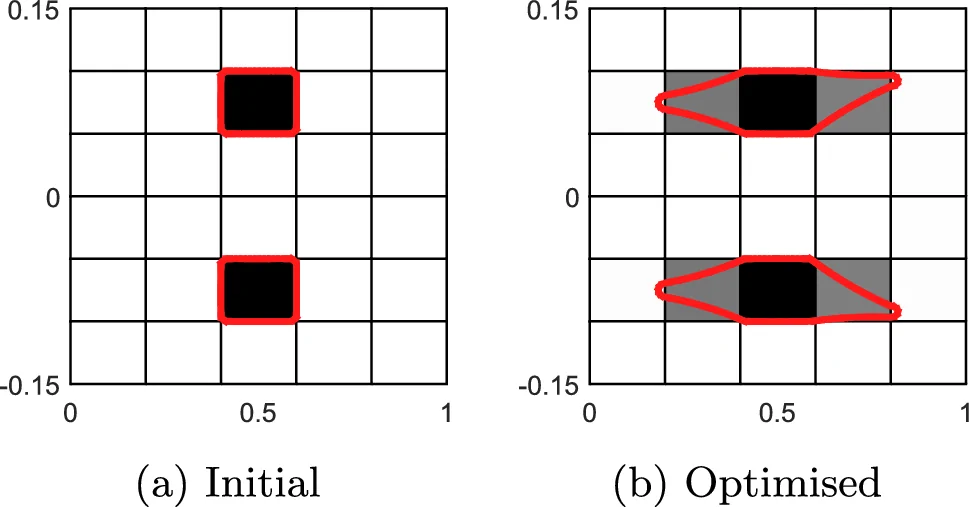
Initial and optimised geometry (red) for the 0.1 spaced case overlaid onto its control mesh. The shading represents $v_t$ in each control cell

**Fig. 31:**
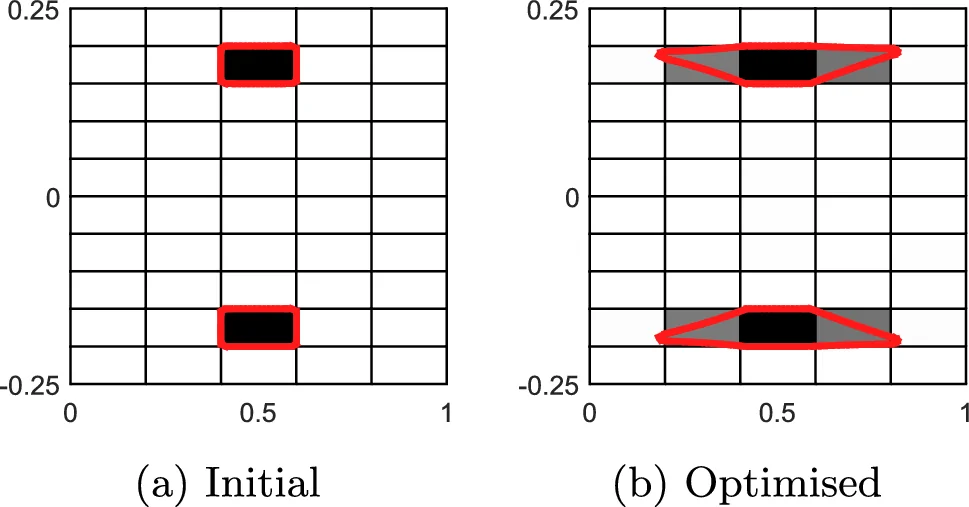
Initial and optimised geometry (red) for the 0.3 spaced case overlaid onto its control mesh. The shading represents $v_t$ in each control cell

To ensure comparable performance across all three cases, the spacing between the objects was changed while maintaining the same physical dimensions of each control cell. This was achieved by adding more control cells between the two objects. As such, the 0.05 spacing case utilised a 5×5 control mesh, Fig. 29a, the 0.1 spacing case a 6×5 control mesh, Fig. 30a, and the 0.3 spacing case a 10×5 control mesh, Fig. 31a. The results of the optimisation of each case are summarised in Table 3 and Figs. 27, 28, 29, 30 and 31.

**Table 3:** Drag coefficients of the objects to be packaged and the optimised geometries for each case

**Spacing**	$\mathbf {0.05}$	$\mathbf {0.10}$	$\mathbf {0.30}$
**Initial ** $\boldsymbol{C_{d0}}$	0.1557	0.1516	0.1512
**Optimised ** $\boldsymbol{C_d}$	0.08474	0.02045	0.01346
$\boldsymbol{C_d} - \boldsymbol{C_{d0}}$ $\boldsymbol{\%}$	-45.56	-86.51	-91.10

As the spacing between the fixed objects increased the optimal geometry changed considerably, with a change in topology occurring between the smallest and second smallest spacings. The 0.05 spacing case resulted in a double-wedge aerofoil like geometry, the 0.1 spacing case resulted in a converging–diverging nozzle like geometry, and the 0.3 spacing case resulted in a Busemann biplane like geometry, Kusunose et al. (2011).

The smallest 0.05 spaced case constructed a single object. This consisted of a double-wedge aerofoil like geometry that maintained supersonic flow over its entire surface, except at the stagnation around the front of the object, Fig. 28a. This single object solution likely arose due to difficulty constructing two objects that did not result in subsonic choked flow between them, and thus a strong bow shock in front of both. This difficulty was due to the implicit limit on the minimum feature size the parameterisation could represent, itself defined by the resolution of the control mesh. Thus, it is possible that a superior two object geometry could be found if a finer control mesh were used, such that the parameterisation could construct a sharper and more accurately positioned front to each object.

The medium 0.1 spaced case constructed a geometry resembling a converging intake duct followed by a diverging nozzle. The inlet region in front of the fixed objects was constructed such that a set of oblique shock waves were formed, which compressed the flow while keeping it fully supersonic though the space between the objects, Fig. 28b. The rear section of the geometry then resembled a slightly over-expanded nozzle, with the Mach number and pressure coefficient across the exit plane averaging 2.072 and -0.06162 respectively, both close to their freestream values. These two features likely ensured the greatest possible mass flux between the objects, thus preventing the formation of a strong bow shock in front of both.

The largest 0.3 spaced case appears to have taken a different approach to the smaller two cases, likely as choking the flow between the objects is less of a concern due to the larger spacing between them. This case has constructed a geometry somewhat similar to a Busemann biplane, exploiting shock-expansion cancelling for reductions in drag. Here, it appears that the shock formed at the front of each object reflects off the expansion at the rear of the flat surface formed on the ‘inside’ of the two constrained $v_t = 1$ control cells, Fig. 28c. This appears to cancel the expansion that would occur here and leads to a higher pressure on the rearmost flat surface of each object, thus eliminating any drag caused by lower than freestream pressure in these regions.

Additionally, in both the 0.1 and 0.3 spaced cases, the optimiser has exploited the down-sampling of the sub-grid from $R_c = 64 \rightarrow R_c = 16$ to construct small regions of ρ=1 sub-grid cells that did not result in the construction of geometry at $R_c = 16$, but that manipulated the position of the geometry in adjacent control cells. This can be seen around the rear section of the 0.1 spaced case, Fig. 30b, and both the front and rear sections of the 0.3 spaced case, Fig. 31b, where in both instances it resulted in a flatter ‘outer’ surface of each pair of objects.

### Topology-inclusive constrained volume drag minimisation at mach 2

To demonstrate the potential benefits of topology-inclusive optimisation for aerodynamic objectives, in this section, the largest volume case from section 6.2 has been repeated using the gradient-free optimisation framework from section 6.3. The optimisation problem was thus defined by Eq. 23.

23 $$\begin{aligned} \begin{aligned} \text {min }&: C_d \\ \text {subject to }&: V_t = 0 \\&: v_t(y> 0) = v_t(y < 0) \\ \end{aligned} \end{aligned}$$

For this case, the geometry was represented using an 8×8 control mesh spanning the region x∈[0,1] y∈[-0.4,0.4]. A symmetry constraint was applied across y=0 using the same method described in section 6.3 to avoid exploration of non-optimal lifting geometries. The volume of the geometry was constrained by scaling all the volume-of-solid parameters by a factor *s* such that $s\sum v_t \cdot V_{ctr} = V_t$, with $V_{ctr}$ the volume of each control cell. The chord of the parameterised geometry was constrained to exactly span the region x∈[0,1] through a shifting and scaling operation defined by Eq. 24. This ensured the chord was always exactly one, while maintaining equal volume to the initial un-scaled geometry thus ensuring that the volume constraint remained always satisfied.

24 $$\begin{aligned} \begin{aligned} s_g =&\max (x - \min (x)) \\ x =&\frac{x - \min (x)}{s_g}\\ y =&s_g y \\ \end{aligned} \end{aligned}$$

**Fig. 32:**
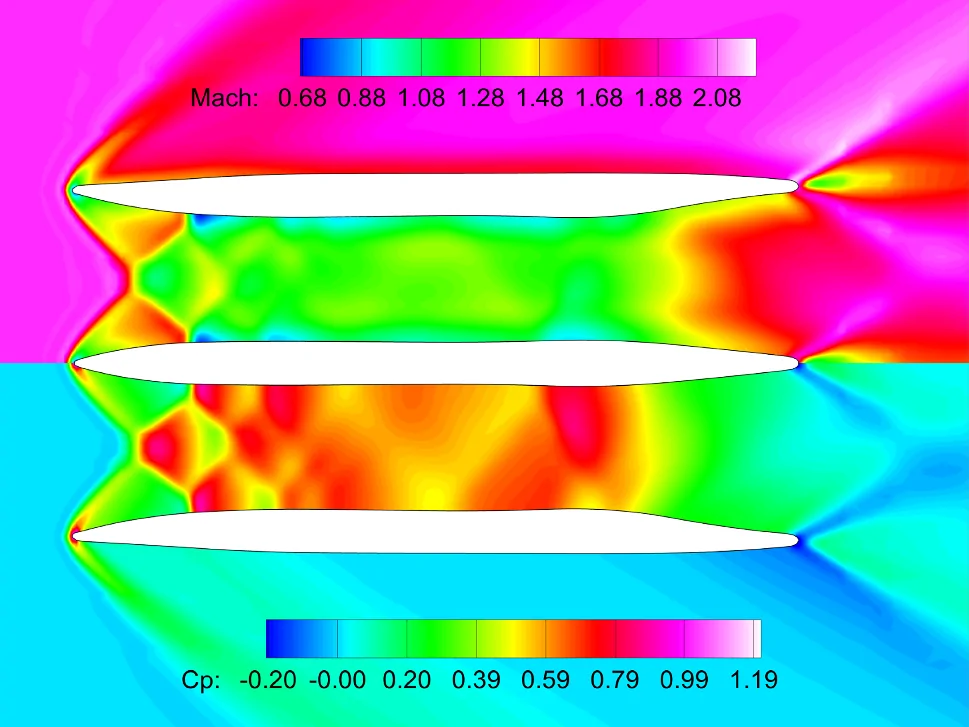
The flow field around the optimised three object geometry

**Fig. 33:**
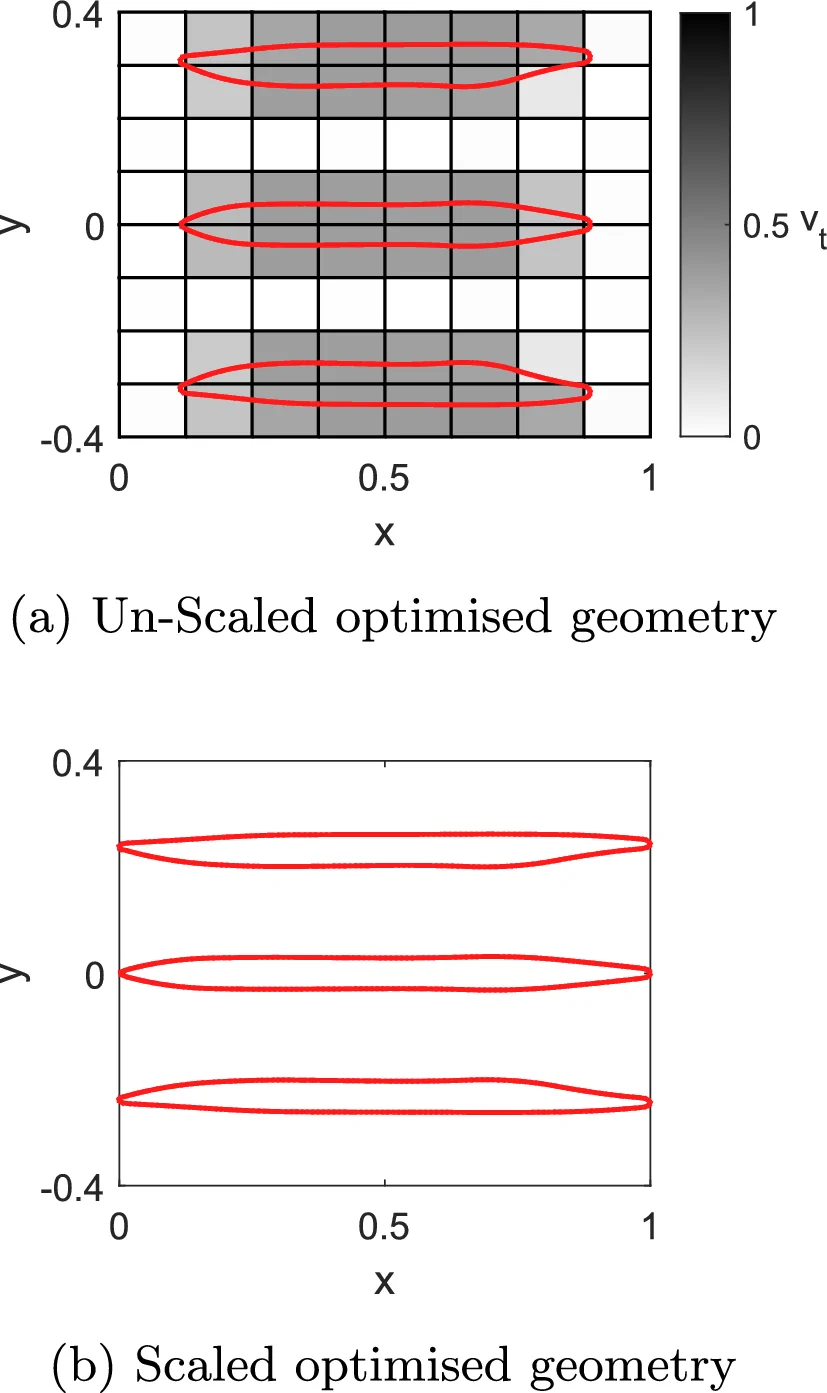
The un-scaled optimised geometry overlaid onto the 8×8 control mesh used to define it, and the geometry scaled using Eq. 24

**Fig. 34:**
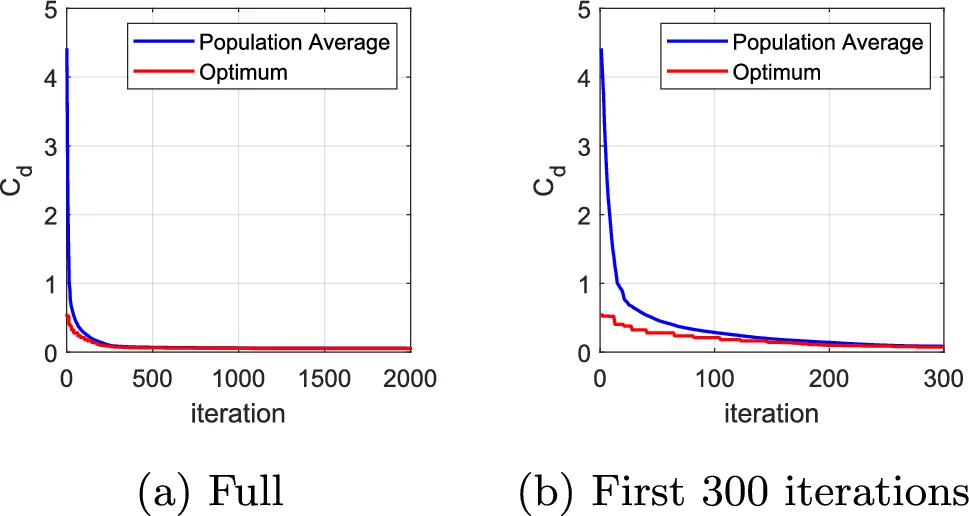
The convergence history of the volume constrained differential evolution optimisation procedure

As both constraints were enforced through the parameterisation, the optimisation itself was unconstrained. Consequently, again to allow exploration of multiple locally optimal geometries of differing topology, the optimisation was performed using the same differential evolution optimiser used in section 6.3. Again following the guidance of Storn and Price (1997), the optimiser was configured using a number of agents approximately 10× the number of design variables. The optimisation was once again terminated when negligible changes in the population average objective value were observed. The results of this optimisation are summarised in Table 4 and Figs 32, 33 and 34.

**Table 4:** The initial population average $C_d$ and the $C_d$ of the optimised geometry

Initial (average)	Optimised	$\boldsymbol{C}_d - \boldsymbol{C}_{d0}$ %
4.4149	0.05044	− 98.86

The optimised geometry consisted of three objects, and appears relatively similar to the result of a similar case of smaller constrained volume attempted previously by Payot et al. (2017). The optimised geometry constructed in this section resulted in a substantial reduction in drag coefficient, of almost 99% compared to the average of the initial randomly sampled population of geometries. This geometry was also constructed relatively rapidly, only taking approximately 500 iterations of the differential evolution process to arise. The optimisation however was run for 2000 iterations in total to ensure no further improvements could be made.

By placing the front and rear of each object near the corner of control cells, the optimiser was able to construct pseudo-sharp features using small $v_t$ in these control cells, Fig. 33a. This resulted in a geometry where a series of reflecting oblique shocks extended through the space between each object, slowing and compressing the flow, while maintaining supersonic flow throughout almost all the space between the objects, Fig. 32. Furthermore, the rear of the geometry took on a nozzle-like appearance in the space between the central object and each outer object. This is similar to the optimised geometry in the 0.1 spacing case in section 6.3. As with that case, these features ensured that the flow did not choke in the space between the objects, and thus allowed the maximum possible mass flux to pass between them preventing formation of a strong bow shock in front of the objects.

Overall, the result of this optimisation was a geometry with a drag coefficient 56.71% smaller than that of the geometry of an equivalent volume constructed in section 6.2 using fixed topology gradient-based optimisation. This significant improvement is the result of including the ability to explore novel topologies within the optimisation, and is likely indicative of the potential improvements that could be achieved across other aerodynamic shape optimisation cases if such an approach were taken. It can also be noted that this improvement was achieved using no control mesh refinement. Hence, it is likely that further improvements could be made to this optimised geometry if this case was run in an optimise-refine type framework similar to that used in section 6.2.

## Conclusion

In this work, the novel cellular automata volume-of-solid (CAVoS) parameterisation has been presented. This method uses a cellular automata scheme coupled to a surface smoothing algorithm to construct geometry of approximately minimum surface area subject to a set of localised contained volume constraints defined by a control mesh in combination with a set of volume-of-solid parameters. The parameterised geometry can be manipulated by changing the contained volume constraints within each cell of the control mesh, defined by each corresponding volume-of-solid parameter. Due to the geometrically abstract nature of these design variables, the parameterisation is capable of representing geometry of arbitrary topology, along with the parameterisation of changes in topology on the same control mesh with only changes to the design variables.

By applying non-uniform control mesh refinement, the parameterisation was shown to be able to re-construct up to 98.3% of the aerofoils within the UIUC aerofoil library to within the Kulfan wind tunnel tolerance. This, however, required on average 75 design variables, many more than most existing parameterisations. To counter the negative effects of this high dimensionality within the context of aerodynamic shape optimisation, an optimise-refine framework can be utilised. This involved refining the control mesh progressively during an optimisation, introducing more design variables only as the geometry approaches the optimum. This methodology has previously been shown to significantly aid the performance of high-dimensional optimisations.

To test this optimise-refine procedure, a set of gradient-based aerodynamic shape optimisation were attempted using it. These involved drag minimisation in supersonic flow under a set of varying equality constraints on the volume of the parameterised geometry. The presented parameterisation was able to recover optimal geometries similar to existing analytically derived optima for each case, with similar but slightly larger drag coefficients. This was the result of a combination of the parameterisations inability to change the active set of control cells during an optimisation, and its inability to represent sharp features without significant control mesh refinement.

Following this, a set of cases to test the utility of the parameterisation for topology-inclusive aerodynamic shape optimisation were attempted. The first set of cases involved the aerodynamic packaging of a set of two rectangular objects at three different spacings in supersonic flow, aiming to minimise the drag coefficient. It was shown that as the objects were moved apart, the parameterisation was able to recover optimal packaging geometries of varying topology. At the smallest spacing, a single object double-wedge aerofoil like geometry was recovered, whereas at the largest spacing a two object Busemann biplane like geometry was recovered. Across all spacings, the optimum packaging geometries reduced the drag coefficient by at least 45.56%, rising to 91.10% at the largest spacing.

Finally, to demonstrate the potential benefits of topology-inclusive aerodynamic shape optimisation, the volume constrained drag minimisation case with the largest volume constraint attempted previously in this work was re-run using a gradient-free optimisation algorithm. Coupled to the topology-inclusive CAVoS parameterisation developed in this work, this allowed exploration and comparison of many locally optimal geometries to find a novel optimal topology. This resulted in a geometry consisting of three objects, that had a drag coefficient 56.71% smaller than the single object optimal geometry constructed previously in this work. This result clearly demonstrates the benefits of the presented parameterisation, and highlights the potential improvements in aerodynamic performance that may be realised through the application of topology-inclusive aerodynamic shape optimisation methods.

## Declarations

### Replication of results

To reproduce any results within this work the corresponding author will, upon request, provide all required geometries and input parameters
